# Machine learning-based prediction of short-term outcomes in aneurysmal subarachnoid hemorrhage: a multicenter study integrating clinical and inflammatory indicators

**DOI:** 10.1186/s12916-025-04523-y

**Published:** 2025-11-29

**Authors:** Weichong Zhou, Cong Peng, Peize Li, Yufei Li, Xingfu Liao, Zhuo Wang, Mingfeng Wang, Yunchong Xiao, Hai Su, Hui Shi

**Affiliations:** 1 Yongchuan Hospital of Chongqing Medical University, Chongqing, 402160 China; 2 First Hospital of Shanxi Medical University, Taiyuan, Shanxi Province 030001 China; 3 Second Hospital of Shanxi Medical University, Taiyuan, Shanxi Province 030001 China; 4 Second Hospital Affiliated to Xiangya School of Medicine, Central South University, Changsha, Hunan Province 410125 China; 5 Department of Geriatrics, Eighth Medical Center, PLA General Hospital, Beijing, 100091 China; 6 Department of Neurosurgery of Yongchuan Hospital of Chongqing Medical University, Chongqing, 402160 China

**Keywords:** Aneurysmal subarachnoid hemorrhage, Inflammatory markers, Interpretable machine learning, Predictive modeling, Visualization

## Abstract

**Background:**

Aneurysmal subarachnoid hemorrhage (aSAH) is a life-threatening cerebrovascular emergency. We built and validated a machine learning model integrating clinical and inflammatory indicators for early risk prediction.

**Methods:**

This multicenter retrospective cohort study included 1,120 aSAH patients admitted between January 2022 and December 2024 across four tertiary hospitals for model development and 326 independent patients from the Second Xiangya Hospital for quasi-external validation. Twenty-eight candidate predictors were evaluated, encompassing clinical grading scales and inflammation- and nutrition-related biomarkers. Continuous variables were discretized into quartile-based categories to enhance interpretability and mitigate outlier effects. Synthetic minority oversampling (SMOTE) addressed outcome imbalance. Feature selection used a two-step process combining the Boruta algorithm and least absolute shrinkage and selection operator (LASSO) regression, with variance inflation factor (VIF) analysis confirming the absence of collinearity. Six supervised algorithms were trained with tenfold cross-validation: logistic regression, neural network, random forest (RF), support vector machine (SVM), gradient boosting machine (GBM), and extreme gradient boosting (XGBoost). Model performance was evaluated by discrimination, calibration, and decision curve analysis, and interpretability was assessed with Shapley additive explanations (SHAP) and Local Interpretable Model-Agnostic Explanations (LIME).

**Results:**

The GBM model achieved the best performance, with an AUC of 0.895 (95% CI: 0.856–0.934) in internal validation and 0.864 (95% CI: 0.822–0.906) in quasi-external validation. Nine predictors were retained: procalcitonin, C-reactive protein-to-lymphocyte ratio (CLR), WFNS grade, systemic immune-inflammation index (SII), prognostic nutritional index (PNI), neutrophil-to-albumin ratio (NAR), Glasgow Coma Scale (GCS), platelet-to-lymphocyte ratio (PLR), and modified Fisher grade. A web-based calculator was implemented for individualized risk prediction.

**Conclusions:**

The GBM-based model enables early prediction of poor short-term outcomes in aSAH, supporting timely clinical decision-making. Prospective multicenter validation is warranted to confirm its generalizability across diverse populations.

**Supplementary Information:**

The online version contains supplementary material available at 10.1186/s12916-025-04523-y.

## Background

Aneurysmal subarachnoid hemorrhage (aSAH) is the third most common type of stroke after cerebral infarction and intracerebral hemorrhage. It is usually caused by rupture of an intracranial aneurysm and is characterized by abrupt onset, high morbidity, and substantial mortality [1]. Although diagnostic imaging and treatment have improved in recent decades [2, 3], aSAH remains a major public health problem, especially in aging populations in East Asia [4, 5]. Some critically ill patients, especially those with high World Federation of Neurosurgical Societies (WFNS) grades or extensive bleeding, can benefit from early aggressive treatment and achieve meaningful recovery [6]. However, many remain at high risk of complications such as cerebral vasospasm, delayed cerebral ischemia (DCI), and neurocognitive impairment, which markedly reduce quality of life and worsen long-term prognosis.

Increasing evidence indicates that inflammation is a key pathological mechanism contributing to secondary neurological injury after aSAH. Hemorrhage rapidly activates the peripheral immune system, inducing the release of proinflammatory cytokines and infiltration of immune cells into the central nervous system. These responses aggravate neuronal damage and hinder functional recovery [7]. Several inflammation-related biomarkers, including the neutrophil-to-lymphocyte ratio (NLR), platelet-to-lymphocyte ratio (PLR), platelet-to-albumin ratio (PAR), systemic immune-inflammation index (SII), prognostic nutritional index (PNI), and C-reactive protein-to-lymphocyte ratio (CLR), have been associated with outcomes in stroke populations [8–11]. These routinely available laboratory measures may allow early risk stratification and support individualized treatment decisions. Accurate prognostication in aSAH is crucial for tailoring therapy and for the rational allocation of medical resources. However, most current predictive models, such as the SAFIRE classification system [12] and the SAHIT model [13], rely mainly on single clinical grading scales and show limited ability to predict early or long-term outcomes. Their restricted use of inflammatory biomarkers further reduces value for early risk stratification and limits applicability to data-driven clinical decision-making [14].

Machine learning (ML) methods are increasingly applied in neurocritical care to improve prognostic modeling. By processing high-dimensional data and capturing complex nonlinear associations, ML algorithms can outperform traditional statistical approaches in predictive accuracy [15, 16]. To address the challenge of limited transparency, explanatory methods such as Shapley Additive Explanations (SHAP) and Local Interpretable Model-Agnostic Explanations (LIME) have been introduced [17, 18]. SHAP provides global estimates of each variable’s contribution to model output, while LIME offers local explanations for individual predictions. Used together, these techniques enhance model transparency and facilitate clinical interpretation. Nonetheless, many ML studies remain limited by single-center data, relatively small sample sizes, and a lack of external validation, which reduces generalizability. In addition, the “black-box” nature of ML models, only partly clarified by SHAP and LIME, continues to restrict their integration into clinical practice [6, 14].

To overcome these limitations, we conducted a multicenter retrospective study using electronic medical records (EMRs) from four tertiary hospitals. Clinical severity scores were combined with inflammation-related biomarkers, and feature selection was performed in two steps using the Boruta algorithm followed by least absolute shrinkage and selection operator (LASSO) regression. Six supervised machine learning models were developed and compared: logistic regression, neural networks, random forest (RF), support vector machine (SVM), gradient boosting machine (GBM), and extreme gradient boosting (XGBoost). Model performance was evaluated in the training cohort and validated in an independent quasi-external validation cohort. The best-performing model was further examined with SHAP analysis and deployed as a publicly accessible web-based prediction tool. This model is intended to enable early individualized risk assessment and to support clinical management of patients with aSAH.

## Methods

### Study design and ethical considerations

This multicenter retrospective cohort study was based on electronic medical records (EMRs) from four tertiary medical centers: Yongchuan Hospital of Chongqing Medical University, the First Hospital of Shanxi Medical University, the Second Hospital of Shanxi Medical University, and the Second Xiangya Hospital of Central South University. Patients admitted between January 1, 2022, and December 31, 2024, were screened for eligibility.

The study was conducted in accordance with the principles of the Declaration of Helsinki and reported following the Strengthening the Reporting of Observational Studies in Epidemiology (STROBE) guidelines. Ethical approval was obtained from the institutional review boards of all participating centers (approval numbers: 2025EC0109, Z0651-01, KYLL-2025–250, and 2025-YX218).

### Study population and eligibility criteria

Patients were eligible if they met all of the following criteria: (1) age ≥ 18 years; (2) admission with a confirmed diagnosis of aSAH secondary to rupture of an intracranial aneurysm, verified by computed tomography angiography (CTA) or digital subtraction angiography (DSA); and (3) receipt of definitive treatment (surgical clipping or endovascular coiling) within 48 h of symptom onset.

Exclusion criteria were: (1) subarachnoid hemorrhage due to non-aneurysmal causes, including trauma, arteriovenous malformation, or arteriovenous fistula; (2) recurrent aSAH or need for a second neurosurgical intervention during hospitalization; (3) in-hospital death; (4) coexisting acute or chronic infections, autoimmune disease, malignancy, uremia, hepatic cirrhosis, chronic cardiac disease, or chronic pulmonary disease; and (5) pre-existing neurological deficits from prior stroke.

### Clinical features

Clinical and laboratory variables were extracted from EMRs of all eligible patients. Baseline variables included age, sex, smoking and alcohol history, and comorbidities (e.g., hypertension, diabetes mellitus). Admission characteristics comprised the Glasgow Coma Scale (GCS) score, WFNS grade, modified Fisher grade, aneurysm location (based on CTA or DSA), and treatment modality (surgical clipping or endovascular coiling). Laboratory tests included routine blood counts, serum albumin, blood glucose, creatinine, D-dimer, fibrinogen, and inflammatory markers such as C-reactive protein (CRP) and procalcitonin.

Inflammation- and nutrition-related indices were calculated using established formulas. These included the prognostic nutritional index (PNI = albumin + 5 × lymphocyte count), neutrophil-to-albumin ratio (NAR), platelet-to-albumin ratio (PAR), neutrophil–lymphocyte–platelet ratio (NLPR), platelet-to-lymphocyte ratio (PLR), monocyte-to-lymphocyte ratio (MLR), systemic immune-inflammation index (SII), systemic inflammation response index (SIRI), C-reactive protein-to-lymphocyte ratio (CLR), and the aggregate index of systemic inflammation (AISI).

### Assessment of study outcomes

The primary outcome was the modified Rankin Scale (mRS) score at hospital discharge. The mRS is a validated tool for assessing functional outcomes after cerebrovascular events, ranging from 0 (no symptoms) to 6 (death) [19]. All assessments were performed within 24 h before discharge by neurologists or rehabilitation physicians according to a standardized protocol.

For model development, mRS scores were dichotomized as follows: favorable outcome, mRS 0 to 2; unfavorable outcome, mRS 3 to 6. This classification has been widely used in studies of stroke and aSAH to represent clinically meaningful thresholds for functional independence [20, 21].

### Statistical analysis

All statistical analyses were conducted using R software (version 4.4.1), SPSS (version 27.0; IBM Corp., Armonk, NY, USA), and Python (version 3.10.4). The distribution of continuous variables was examined by visual inspection of histograms and the Shapiro–Wilk test. Variables with non-normal distributions were summarized as medians with interquartile ranges (IQRs), and categorical variables were presented as counts and percentages. Between-group comparisons were performed using the chi-square test for categorical variables. The Mann–Whitney U or Kruskal–Wallis H tests were applied for continuous or ordinal variables. Correlations between continuous variables were assessed with Spearman’s rank correlation coefficients.

To reduce potential instability from high variability in continuous predictors, selected variables were discretized into ordered categories using quartile-based binning. When class imbalance was present, the synthetic minority oversampling technique (SMOTE) was applied to address the imbalance and improve the prediction of the minority outcome class. Feature selection was performed in two steps. The Boruta algorithm was first applied to identify candidate predictors, and least absolute shrinkage and selection operator (LASSO) regression was then used for dimensionality reduction and to control multicollinearity. Variance inflation factors (VIFs) were additionally calculated to evaluate collinearity among the selected predictors.

Six supervised machine learning algorithms were developed to classify functional outcomes: logistic regression (LR), random forest (RF), support vector machine (SVM), gradient boosting machine (GBM), extreme gradient boosting (XGBoost), and neural networks (NN). All models were trained in the development cohort using tenfold cross-validation to evaluate stability and generalizability. Performance metrics included accuracy, the area under the receiver operating characteristic curve (AUC), sensitivity, specificity, F1 score, and Brier score. Calibration was assessed using Brier scores and calibration curves, and the calibration intercept and slope were estimated. Decision-curve analysis (DCA) was performed with the ggDCA package to evaluate net clinical benefit across a range of threshold probabilities.

To enhance interpretability, Shapley Additive Explanations (SHAP) and Local Interpretable Model-Agnostic Explanations (LIME) were applied. SHAP, implemented with the shapviz package, quantified the contribution of each predictor to both individual predictions and overall model output. LIME, implemented in Python with the lime library, provided case-level explanations by approximating local decision boundaries. Together, these methods provided complementary perspectives on global feature importance and local prediction logic.

## Results

### Patient cohorts and baseline characteristics

**Fig. 1:**
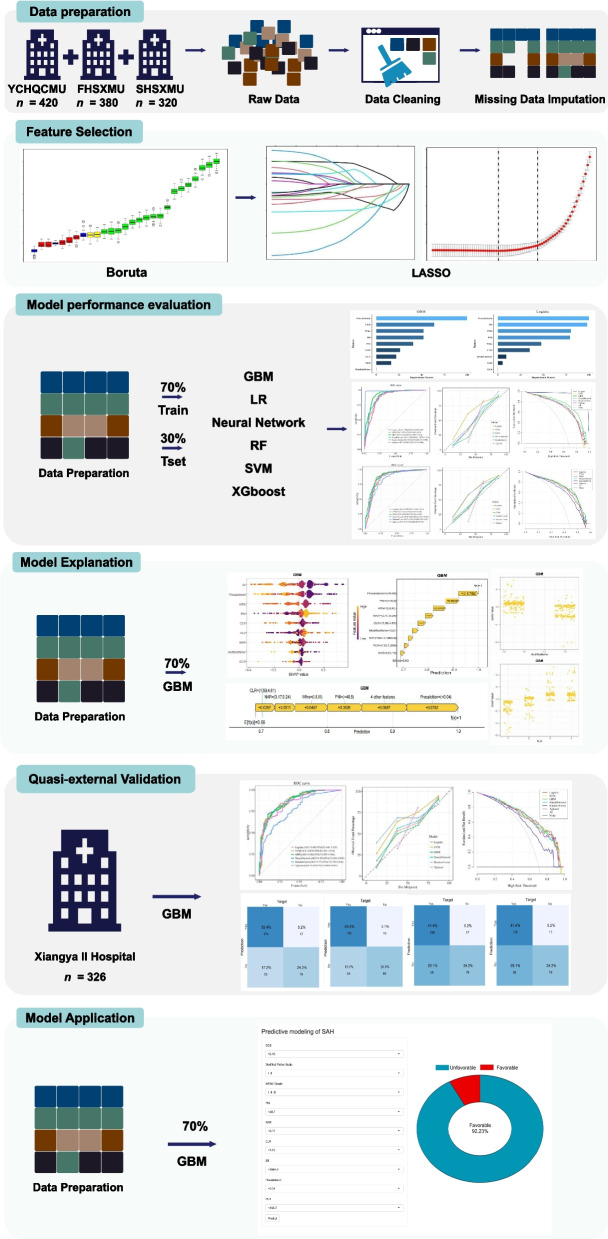
Workflow of model development and validation. The workflow illustrates data preprocessing, feature selection using Boruta and LASSO, model training across six algorithms, external validation, and online deployment. Training data were obtained from three centers (YCHCQMU, FHSMU, SHSMU), and quasi-external validation cohort relied on data from the Second Xiangya Hospital. The GBM model, which demonstrated optimal performance, was implemented as a web-based risk calculator

Figure 1 shows the overall study workflow. A total of 1,446 patients with aSAH were recruited from four tertiary medical centers. Of these, 1,120 patients from three centers were randomly assigned to the training cohort (*n* = 784) and the internal test cohort (*n* = 336) in a 7:3 ratio. The remaining 326 patients from the Second Xiangya Hospital of Central South University comprised the quasi-external validation cohort.

Baseline demographic and clinical characteristics of the three cohorts are summarized in Table 1. Overall, 69.2% of patients (*n* = 1,001) had a favorable short-term functional outcome (mRS 0 to 2), whereas 30.8% (*n* = 445) had an unfavorable outcome (mRS 3 to 6). The proportion of favorable outcomes differed significantly across cohorts (*P* = 0.029), which may reflect variation in disease severity or treatment strategies. Female patients accounted for 62.5% of the total cohort, with significant variation in sex distribution across cohorts (*P* = 0.005). Admission GCS scores, WFNS grades, aneurysm location, and treatment modality also differed significantly between cohorts (all *P* < 0.05).

**Table 1:** Baseline characteristics of the study population across training, internal test, and external validation cohorts

**Variables**	**Total (*n*= 1446)**	**test (*n*= 336)**	**train*(n*= 784)**	**Valid (*n*= 326)**	**P**
mRS, n(%)					**0.029**
(3,6)	445 (30.77)	98 (29.17)	263 (33.55)	84 (25.77)	
(0,2)	1001 (69.23)	238 (70.83)	521 (66.45)	242 (74.23)	
Sex, n(%)					**0.005**
Male	542 (37.48)	140 (41.67)	304 (38.78)	98 (30.06)	
Female	904 (62.52)	196 (58.33)	480 (61.22)	228 (69.94)	
Age, n(%)					0.646
(<50)	336 (23.24)	83 (24.70)	175 (22.32)	78 (23.93)	
(50,58)	362 (25.03)	83 (24.70)	203 (25.89)	76 (23.31)	
(58,67)	381 (26.35)	78 (23.21)	217 (27.68)	86 (26.38)	
(>67)	367 (25.38)	92 (27.38)	189 (24.11)	86 (26.38)	
Smoke, n(%)					0.710
No	1123 (77.66)	262 (77.98)	603 (76.91)	258 (79.14)	
Yes	323 (22.34)	74 (22.02)	181 (23.09)	68 (20.86)	
Drink, n(%)					0.498
No	1181 (81.67)	275 (81.85)	633 (80.74)	273 (83.74)	
Yes	265 (18.33)	61 (18.15)	151 (19.26)	53 (16.26)	
GCS, n(%)					**0.032**
(13,15)	838 (57.95)	204 (60.71)	455 (58.04)	179 (54.91)	
(8,12)	494 (34.16)	104 (30.95)	259 (33.04)	131 (40.18)	
(3,7)	114 (7.88)	28 (8.33)	70 (8.93)	16 (4.91)	
Hypertension, n(%)					0.863
No	706 (48.82)	161 (47.92)	382 (48.72)	163 (50.00)	
Yes	740 (51.18)	175 (52.08)	402 (51.28)	163 (50.00)	
Diabets, n(%)					0.177
No	1342 (92.81)	313 (93.15)	734 (93.62)	295 (90.49)	
Yes	104 (7.19)	23 (6.85)	50 (6.38)	31 (9.51)	
Hunthess, n(%)					**<.001**
(I,II)	895 (61.89)	208 (61.90)	457 (58.29)	230 (70.55)	
(III,IV,V)	551 (38.11)	128 (38.10)	327 (41.71)	96 (29.45)	
Modifiedfisher, n(%)					0.600
(I,II)	873 (60.37)	207 (61.61)	464 (59.18)	202 (61.96)	
(III,IV)	573 (39.63)	129 (38.39)	320 (40.82)	124 (38.04)	
WFNS, n(%)					0.394
(I,II,III)	880 (60.86)	212 (63.10)	479 (61.10)	189 (57.98)	
(IV,V)	566 (39.14)	124 (36.90)	305 (38.90)	137 (42.02)	
Aneurysmlocation, n(%)					**<.001**
Anterior Circulation	1278 (88.38)	319 (94.94)	738 (94.13)	221 (67.79)	
Posterior Circulation	168 (11.62)	17 (5.06)	46 (5.87)	105 (32.21)	
Surgicalmethod, n(%)					**0.004**
Interventional treatment	935 (64.66)	192 (57.14)	522 (66.58)	221 (67.79)	
Clipping	511 (35.34)	144 (42.86)	262 (33.42)	105 (32.21)	
Ddimer, n(%)					0.284
(<0.5)	358 (24.76)	69 (20.54)	209 (26.66)	80 (24.54)	
(0.5,1.1)	362 (25.03)	84 (25.00)	197 (25.13)	81 (24.85)	
(1.1,2.6)	364 (25.17)	84 (25.00)	197 (25.13)	83 (25.46)	
(>2.6)	362 (25.03)	99 (29.46)	181 (23.09)	82 (25.15)	
Albumin, n(%)					0.329
(<35.2)	330 (22.82)	76 (22.62)	173 (22.07)	81 (24.85)	
(35.2,39.5)	383 (26.49)	98 (29.17)	207 (26.40)	78 (23.93)	
(39.5,42.6)	407 (28.15)	86 (25.60)	237 (30.23)	84 (25.77)	
(>42.6)	326 (22.54)	76 (22.62)	167 (21.30)	83 (25.46)	
Fibrinogen, n(%)					**<.001**
(<2.45)	290 (20.06)	76 (22.62)	183 (23.34)	31 (9.51)	
(2.45,2.95)	338 (23.37)	94 (27.98)	183 (23.34)	61 (18.71)	
(2.95,3.51)	411 (28.42)	83 (24.70)	222 (28.32)	106 (32.52)	
(>3.51)	407 (28.15)	83 (24.70)	196 (25.00)	128 (39.26)	
PNI, n(%)					**<.001**
(<40.7)	265 (18.33)	58 (17.26)	128 (16.33)	79 (24.23)	
(40.7,45.0)	370 (25.59)	69 (20.54)	186 (23.72)	115 (35.28)	
(45.0,48.8)	359 (24.83)	95 (28.27)	193 (24.62)	71 (21.78)	
(>48.8)	452 (31.26)	114 (33.93)	277 (35.33)	61 (18.71)	
NAR, n(%)					**0.006**
(<0.17)	374 (25.86)	83 (24.70)	209 (26.66)	82 (25.15)	
(0.17,0.24)	481 (33.26)	120 (35.71)	280 (35.71)	81 (24.85)	
(0.17,0.24)	296 (20.47)	68 (20.24)	147 (18.75)	81 (24.85)	
(>0.31)	295 (20.40)	65 (19.35)	148 (18.88)	82 (25.15)	
PAR, n(%)					0.385
(<4.11)	356 (24.62)	73 (21.73)	203 (25.89)	80 (24.54)	
(4.11,5.11)	359 (24.83)	96 (28.57)	189 (24.11)	74 (22.70)	
(5.11,6.24)	356 (24.62)	81 (24.11)	198 (25.26)	77 (23.62)	
(>6.24)	375 (25.93)	86 (25.60)	194 (24.74)	95 (29.14)	
NLPR, n(%)					**<.001**
(<0.03)	329 (22.75)	72 (21.43)	207 (26.40)	50 (15.34)	
(0.03,0.05)	353 (24.41)	96 (28.57)	185 (23.60)	72 (22.09)	
(0.05,0.08)	390 (26.97)	74 (22.02)	206 (26.28)	110 (33.74)	
(>0.08)	374 (25.86)	94 (27.98)	186 (23.72)	94 (28.83)	
PLR, n(%)					0.837
(<133.7)	357 (24.69)	78 (23.21)	200 (25.51)	79 (24.23)	
(133.7,208)	363 (25.10)	87 (25.89)	194 (24.74)	82 (25.15)	
(208,295)	359 (24.83)	78 (23.21)	202 (25.77)	79 (24.23)	
(>295)	367 (25.38)	93 (27.68)	188 (23.98)	86 (26.38)	
MLR, n(%)					0.297
(<0.33)	368 (25.45)	81 (24.11)	201 (25.64)	86 (26.38)	
(0.33,0.52)	460 (31.81)	107 (31.85)	235 (29.97)	118 (36.20)	
(0.52,0.88)	334 (23.10)	74 (22.02)	192 (24.49)	68 (20.86)	
(>0.88)	284 (19.64)	74 (22.02)	156 (19.90)	54 (16.56)	
SIRI, n(%)					0.068
(<2.57)	360 (24.90)	81 (24.11)	197 (25.13)	82 (25.15)	
(2.57,5.08)	452 (31.26)	108 (32.14)	263 (33.55)	81 (24.85)	
(5.08,9.68)	343 (23.72)	80 (23.81)	182 (23.21)	81 (24.85)	
(>9.68)	291 (20.12)	67 (19.94)	142 (18.11)	82 (25.15)	
CLR, n(%)					**0.038**
(<1.59)	483 (33.40)	108 (32.14)	282 (35.97)	93 (28.53)	
(1.59,4.81)	479 (33.13)	115 (34.23)	254 (32.40)	110 (33.74)	
(4.81,18.4)	266 (18.40)	65 (19.35)	145 (18.49)	56 (17.18)	
(>18.4)	218 (15.08)	48 (14.29)	103 (13.14)	67 (20.55)	
SII, n(%)					**<.001**
(<1084.4)	472 (32.64)	105 (31.25)	285 (36.35)	82 (25.15)	
(1084.4,1860.8)	431 (29.81)	107 (31.85)	243 (30.99)	81 (24.85)	
(1860.8,3037.3)	303 (20.95)	71 (21.13)	151 (19.26)	81 (24.85)	
(>3037.3)	240 (16.60)	53 (15.77)	105 (13.39)	82 (25.15)	
AISI, n(%)					**0.008**
(<488.4)	400 (27.66)	86 (25.60)	197 (25.13)	117 (35.89)	
(488.4,979.0)	401 (27.73)	88 (26.19)	225 (28.70)	88 (26.99)	
(979.1863.1)	343 (23.72)	87 (25.89)	197 (25.13)	59 (18.10)	
(>1863.1)	302 (20.89)	75 (22.32)	165 (21.05)	62 (19.02)	
Procalcitonin, n(%)					**<.001**
(<0.04)	539 (37.28)	136 (40.48)	322 (41.07)	81 (24.85)	
(0.04,0.12)	347 (24.00)	73 (21.73)	195 (24.87)	79 (24.23)	
(0.12,0.43)	283 (19.57)	59 (17.56)	140 (17.86)	84 (25.77)	
(>0.43)	277 (19.16)	68 (20.24)	127 (16.20)	82 (25.15)	
Sugar, n(%)					0.415
(<6.5)	350 (24.20)	76 (22.62)	193 (24.62)	81 (24.85)	
(6.5,7.4)	353 (24.41)	90 (26.79)	183 (23.34)	80 (24.54)	
(7.4,8.8)	376 (26.00)	98 (29.17)	198 (25.26)	80 (24.54)	
(>8.8)	367 (25.38)	72 (21.43)	210 (26.79)	85 (26.07)	
Creatinine, n(%)					0.873
(<46)	343 (23.72)	84 (25.00)	188 (23.98)	71 (21.78)	
(46,55)	359 (24.83)	77 (22.92)	194 (24.74)	88 (26.99)	
(55,67)	374 (25.86)	86 (25.60)	207 (26.40)	81 (24.85)	
(>67)	370 (25.59)	89 (26.49)	195 (24.87)	86 (26.38)	
Hb, n(%)					0.747
(<116.0)	352 (24.34)	88 (26.19)	183 (23.34)	81 (24.85)	
(116.0,128.0)	353 (24.41)	75 (22.32)	199 (25.38)	79 (24.23)	
(128.0,140.0)	362 (25.03)	77 (22.92)	201 (25.64)	84 (25.77)	
(>140.0)	379 (26.21)	96 (28.57)	201 (25.64)	82 (25.15)	
RBC, n(%)					0.829
(<3.75)	356 (24.62)	82 (24.40)	193 (24.62)	81 (24.85)	
(3.75,4.15)	366 (25.31)	87 (25.89)	197 (25.13)	82 (25.15)	
(4.15,4.56)	352 (24.34)	72 (21.43)	200 (25.51)	80 (24.54)	
(>4.56)	372 (25.73)	95 (28.27)	194 (24.74)	83 (25.46)	

Data are presented as n (%). Variables were grouped according to clinical criteria or quartile distribution. P values were calculated using Pearson’s chi-square test or Fisher’s exact test for categorical variables. A two-sided *P* < 0.05 was considered statistically significant.

*mRS* modified Rankin Scale, *GCS* Glasgow Coma Scale, *WFNS* World Federation of Neurosurgical Societies, *PNI* prognostic nutritional index, *NAR* neutrophil-to-albumin ratio, *NLPR* neutrophil/(lymphocyte × platelet) ratio, *CLR* C-reactive protein-to-lymphocyte ratio, *SII* systemic immune-inflammation index, *AISI* aggregate index of systemic inflammation, *PCT* procalcitonin; *Hb* hemoglobin, *RBC* red blood cell count

Several inflammation- and nutrition-related laboratory markers, including fibrinogen, PNI, NAR, NLPR, SII, AISI, and procalcitonin, also differed significantly across cohorts (all *P *< 0.05). These differences may reflect variation in disease burden, timing of admission, or center-specific management. Despite this heterogeneity, the main clinical and laboratory features remained sufficiently balanced, providing a reasonable basis for model development and validation.

### Correlation analysis of predictive variables

**Fig. 2:**
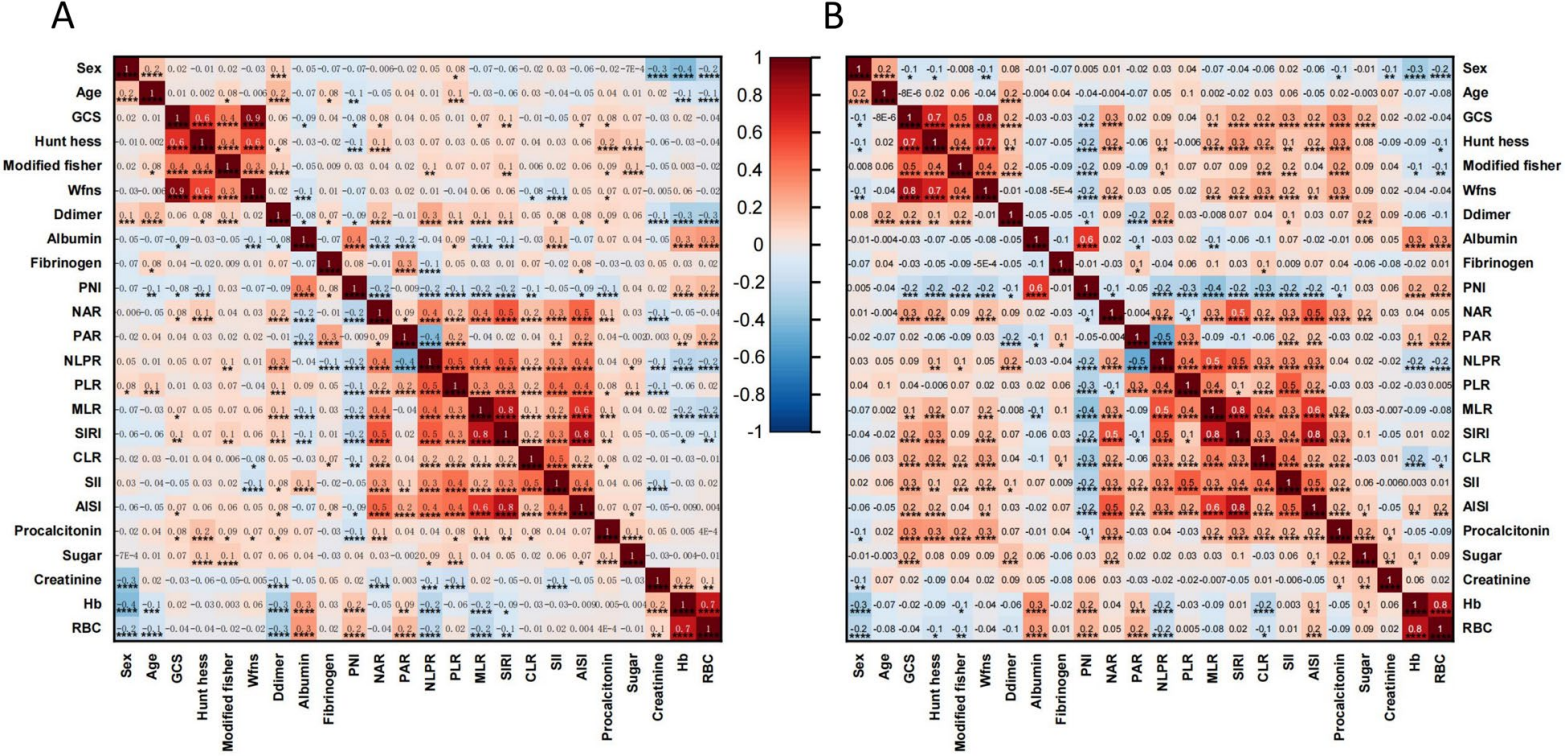
Spearman correlation heatmaps of candidate variables in the training cohort. **A**-**B** Spearman correlation matrices for patients with favorable (mRS 0-2) and unfavorable (mRS 3-6) outcomes. Color denotes correlation strength and direction (blue: positive, red: negative); darker shades represent stronger correlations

Spearman correlation analysis was performed separately in patients with favorable and unfavorable outcomes to evaluate relationships among predictive variables and assess potential multicollinearity. Correlation heatmaps for both subgroups are shown in Fig. 2.

In the favorable outcome group (mRS 0 to 2), most inflammatory biomarkers demonstrated low to moderate positive correlations. For example, NLPR correlated moderately with NAR (R = 0.40, *P* < 0.001) and SIRI (R = 0.50, *P* < 0.001), and weakly with CLR (R = 0.20, *P* < 0.001). PNI correlated negatively with SIRI (R = −0.20, *P* < 0.001) and with Hunt-Hess grade (R = −0.10, *P* = 0.001), suggesting that poorer nutritional status was linked to greater inflammatory burden and higher disease severity.

### Feature selection and variable importance ranking

A total of 28 candidate predictors were considered, including clinical severity scores, laboratory parameters, and derived inflammatory biomarkers. Continuous variables were discretized into quartile-based categories. This binning approach improved interpretability during feature selection and reduced the influence of extreme values, thereby enhancing associations with outcomes (Additional file 2: Fig S1).

**Fig. 3:**
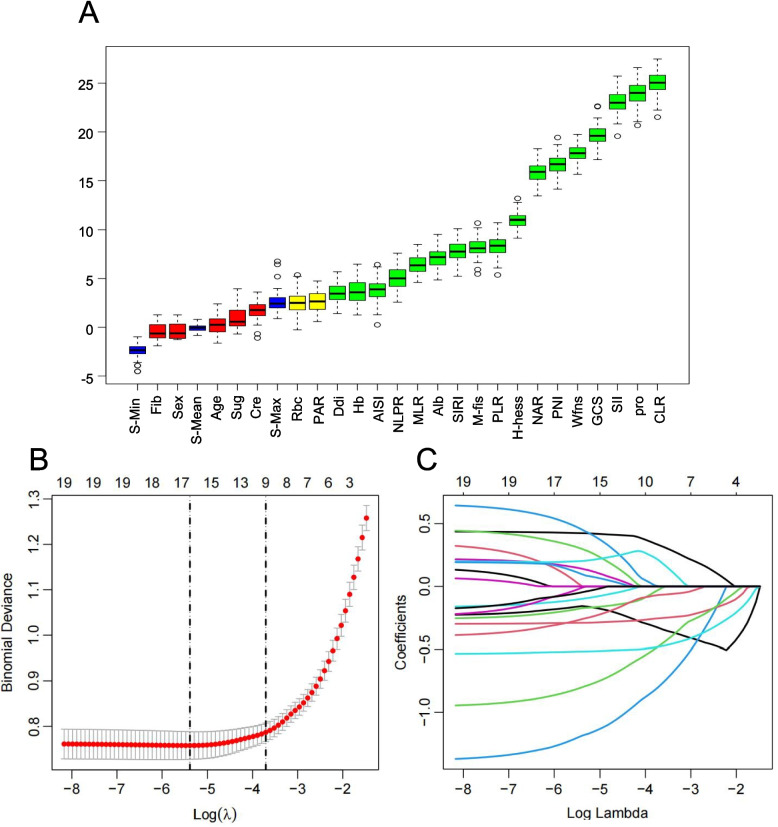
Feature selection process and final predictor selection. **A** Feature importance identified by the Boruta algorithm (green: confirmed, red: rejected, yellow: tentative). **B** Coefficient paths of LASSO regression across varying log(lambda) values. **C** Cross-validation curve used to determine the optimal penalty parameter in LASSO. Nine predictors were ultimately retained at the λ value that minimized binomial deviance in 10-fold cross-validation

To identify the most relevant predictors, a two-step feature selection strategy was used. First, the Boruta algorithm, a wrapper method based on random forest classifiers, screened candidate variables and retained 19 features (Fig. 3A). Next, LASSO regression was applied to reduce dimensionality and address multicollinearity. The optimal penalty parameter (λ) was determined by tenfold cross-validation, and nine predictors were finally selected: GCS score, modified Fisher grade, WFNS grade, PNI, NAR, CLR, SII, procalcitonin, and PLR (Fig. 3B and C).

**Fig. 4:**
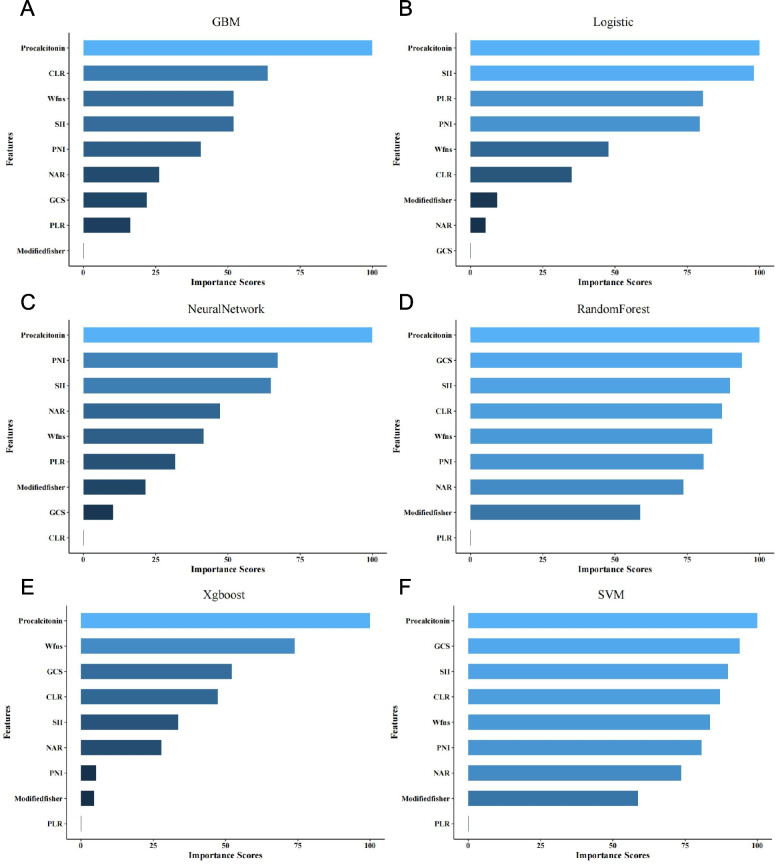
Top-ranked predictors across different machine learning models. The figure depicts the ten most influential features identified by six machine learning algorithms: GBM, XGBoost, random forest (RF), support vector machine (SVM), logistic regression (LR), and neural network (NN). Feature importance was derived using metrics specific to each model (e.g., standardized coefficients for LR, Gini importance for RF).

Collinearity analysis was then performed to ensure that the selected predictors were not linearly dependent. All VIFs were < 5, indicating no evidence of multicollinearity (Additional file 1: Supplementary Table S1). Feature importance rankings were subsequently generated for the six machine learning models using their respective internal measures (e.g., Gini importance for random forest, absolute coefficient magnitude for logistic regression). Across models, procalcitonin, SII, and GCS consistently ranked among the top predictors, highlighting their close association with early functional outcomes (Fig. 4).

### Model performance comparison

**Fig. 5:**
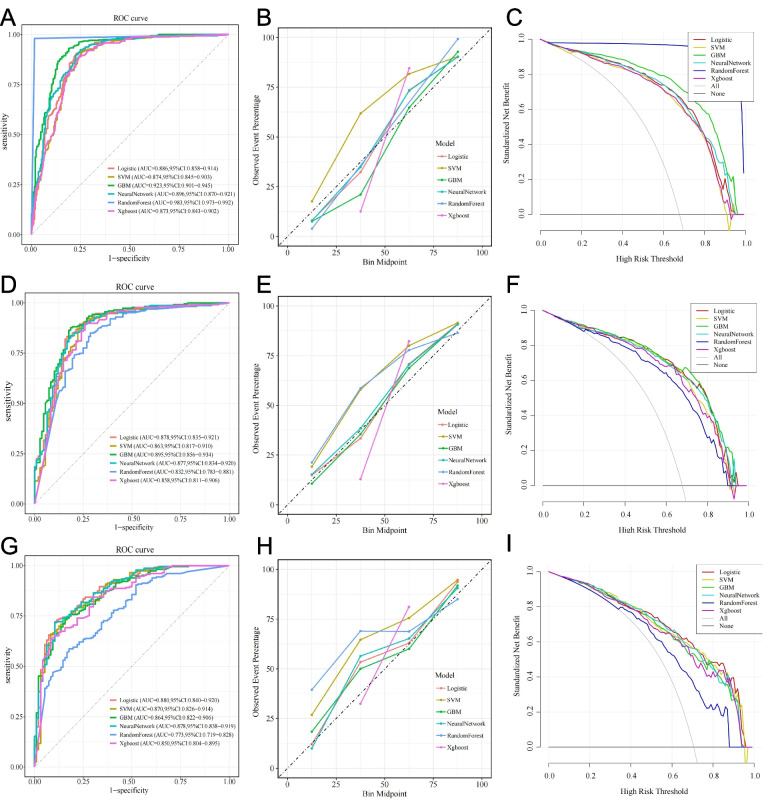
**A**-**C** Receiver operating characteristic (ROC) curves. **D**-**F** Calibration plots. **G**-**I** Decision curve analysis (DCA). Left, middle, and right columns correspond to the training, internal test, and quasi-external validation cohorts

Model performance was assessed in the training, internal test, and quasi-external validation cohorts, as well as by tenfold cross-validation, using multiple classification metrics. In the training cohort, the RF model achieved the best performance, with an AUC of 0.983 (95% CI: 0.973–0.992), the lowest Brier score (0.018), and high values for accuracy (0.982), sensitivity (0.981), specificity (0.984), precision (0.992), and F1 score (0.987) (Fig. 5A-B, Table 2). Decision curve analysis (DCA) showed that the RF model yielded the greatest net clinical benefit across the full range of threshold probabilities (Fig. 5C). However, in tenfold cross-validation, the RF model showed marked variability across folds, consistent with overfitting and reduced AUC performance. In contrast, the GBM model maintained stable performance, with an AUC of 0.87 (95% CI: 0.86–0.88), indicating greater robustness and generalizability (Additional file 3: Figure S2).

**Table 2:** Performance of six machine learning models in the training, internal test, and external validation sets

**Data set**	**Model**	**Accuracy**	**Sensitivity**	**Specificity**	**Precision**	**F1** **Score**	**Brier** **score**	**C-index**	**Dxy**	**R2**	**Intercept**	**Slope**
Train	LR	0.861	0.902	0.775	0.894	0.898	0.109	0.886	0.772	0.428	0	1
	SVM	0.860	0.914	0.747	0.884	0.898	0.128	0.874	0.747	0.379	0.67	0.68
	GBM	0.892	0.930	0.810	0.912	0.921	0.089	0.923	0.847	0.519	−0.18	1.19
	NeuralNetwork	0.869	0.925	0.751	0.886	0.905	0.106	0.896	0.791	0.437	−0.07	1.11
	RF	0.982	0.981	0.984	0.992	0.987	0.018	0.983	0.965	0.867	0.83	0.29
	XGBoost	0.847	0.887	0.763	0.887	0.887	0.248	0.873	0.745	0.394	−0.39	1.33
Test	LR	0.833	0.833	0.833	0.913	0.871	0.118	0.878	0.755	0.39	0.162	0.916
	SVM	0.854	0.903	0.750	0.884	0.893	0.133	0.863	0.727	0.365	0.696	0.636
	GBM	0.857	0.877	0.815	0.909	0.892	0.109	0.895	0.79	0.421	0.093	0.941
	NeuralNetwork	0.830	0.837	0.815	0.905	0.870	0.119	0.877	0.753	0.382	0.157	0.965
	RF	0.806	0.850	0.713	0.862	0.856	0.159	0.832	0.664	0.28	0.425	0.157
	XGBoost	0.848	0.899	0.741	0.879	0.889	0.248	0.858	0.717	0.35	−0.289	1.352
Valid	LR	0.773	0.722	0.896	0.943	0.818	0.125	0.88	0.76	0.352	0.295	1.009
	SVM	0.773	0.748	0.833	0.915	0.823	0.152	0.87	0.74	0.329	0.984	0.752
	GBM	0.776	0.757	0.823	0.911	0.827	0.133	0.864	0.728	0.309	0.294	0.983
	NeuralNetwork	0.770	0.717	0.896	0.943	0.815	0.125	0.878	0.757	0.339	0.209	1.03
	RF	0.656	0.587	0.823	0.888	0.707	0.204	0.773	0.546	0.149	0.661	0.154
	XGBoost	0.718	0.635	0.917	0.948	0.76	0.248	0.85	0.699	0.3	−0.003	1.085

*LR* logistic regression, *SVM* support vector machine, *GBM* gradient boosting machine, *NN* neural network, *RF* random forest, *AUC* area under the curve

In the internal test cohort, the GBM model provided the most stable results, with an AUC of 0.895 (95% CI: 0.856–0.934) and a Brier score of 0.109 (Fig. 5D-E). The RF model remained effective but had a lower AUC and a higher Brier score (0.141), again suggesting overfitting. Logistic regression and SVM performed less well across most metrics. DCA confirmed that the GBM model maintained net clinical benefit in the test cohort (Fig. 5F). Calibration curves for the training and test cohorts further demonstrated the better generalizability of the GBM model (Additional file 4:Figure S3). In the quasi-external validation cohort, the GBM model continued to perform well, with an AUC of 0.864 (95% CI: 0.822–0.906) and a Brier score of 0.133 (Figs. 5G-H). Logistic regression (AUC = 0.880), neural network (AUC = 0.878), and SVM (AUC = 0.870) showed slightly higher AUCs, but the GBM model had better calibration and clinical utility, as supported by its favorable DCA curve (Fig. 5I).

**Fig. 6:**
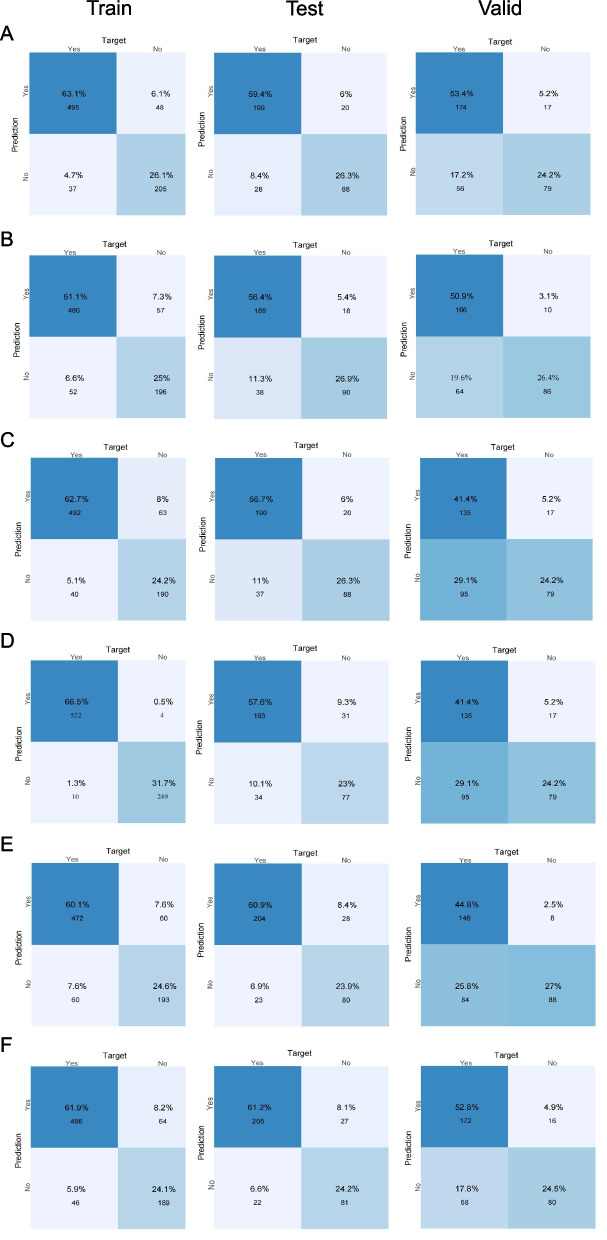
Confusion matrices of six machine learning models across three datasets. Panels **A**-**F** correspond to the following models: (**A**) GBM, (**B**) LR, (**C**) NN, (**D**) RF, (**E**) XGBoost, and (**F**) SVM. Each panel presents true positives (TP), false positives (FP), true negatives (TN), and false negatives (FN), enabling direct comparison of classification accuracy and error distribution across models

To evaluate robustness under class imbalance, the training data were resampled using SMOTE. After SMOTE adjustment, the GBM model maintained comparable performance, with stable AUCs (training cohort: 0.964, 95% CI: 0.954–0.973; test cohort: 0.886, 95% CI: 0.845–0.927) and good calibration. DCA also showed that the SMOTE-adjusted GBM model provided additional net clinical benefit across a broader range of thresholds (Additional file 5:Figure S4). Confusion matrix analysis supported the generalizability of the GBM model. In the test cohort, the GBM classifier achieved a high proportion of true positives and true negatives with relatively few misclassifications (Fig. 6A). By contrast, the RF model produced more false positives in both the test and validation cohorts (Fig. 6D), consistent with weaker generalizability.

Overall, the GBM model had the best calibration among all models, with more favorable intercepts, slopes, and Brier scores, supporting its reliability for use in clinical settings.

### Comparison of model performance with different feature combinations

The discriminatory performance of models constructed with different feature sets was compared using the DeLong test for ROC curves and DCA. The AUC difference between ModA (GCS score, modified Fisher grade, WFNS grade, PNI, NAR, CLR, SII, procalcitonin, and PLR) and ModB (GCS score, modified Fisher grade, WFNS grade) was statistically significant (*p* < 0.001). The difference between ModA and ModC (PNI, NAR, CLR, SII, procalcitonin, and PLR) was also significant (*p* = 0.022), indicating that ModA provided better discrimination for outcome prediction (Additional file 6: Figure S5).

### Model interpretability

**Fig. 7:**
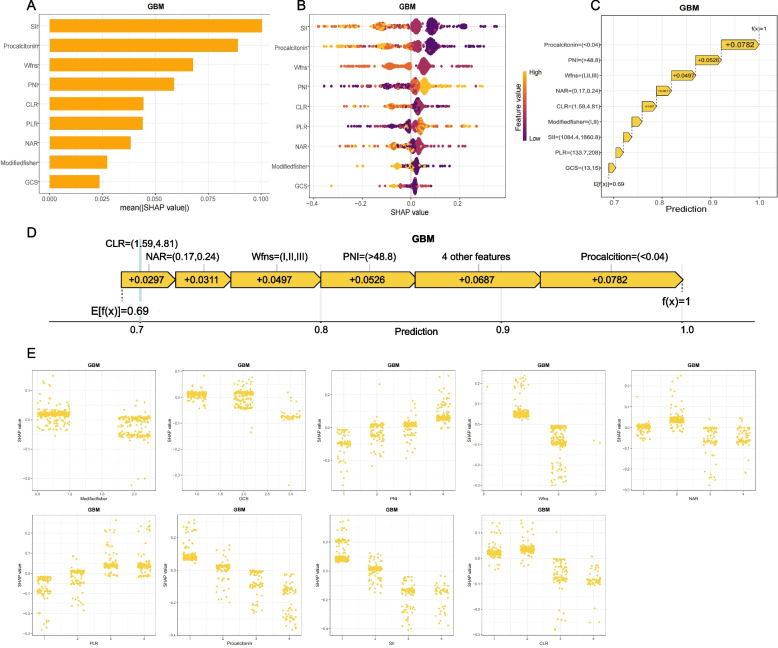
SHAP-based interpretability visualizations for the GBM model. **A** SHAP bar plot displaying mean absolute SHAP values, representing global feature importance. **B** SHAP beeswarm plot depicting SHAP value distributions across all samples, with color denoting original feature values. **C** SHAP waterfall plot demonstrating how the prediction for a representative patient progressed from the base value (E[f(x)]) to the final output (f(x)). **D** SHAP force plot visualizing the positive and negative contributions of predictors for the same sample. **E** SHAP partial dependence-like plots presenting the marginal effects of selected variables, highlighting nonlinear or segmented patterns. Together, these visualizations enhance interpretability and provide a transparent account of variable contributions

To assess interpretability of the final predictive model, SHAP analysis was applied to the GBM classifier. Global importance was evaluated using summary plots of mean absolute SHAP values, which ranked predictors by their overall contributions. As shown in Fig. 7A, SII, procalcitonin, WFNS grade, PNI, and CLR were the most influential variables, suggesting that inflammatory markers and severity scores were the main drivers of model predictions. The beeswarm plot (Fig. 7B) illustrated the distribution and direction of each feature’s effect. Higher values of SII and CLR, together with higher WFNS and modified Fisher grades, were associated with greater risk of poor outcome, whereas higher PNI and lower procalcitonin were associated with favorable outcomes. The color gradient and spread of SHAP values showed how individual predictors affected model output.

Local interpretability was examined using SHAP waterfall and force plots (Fig. 7C–E), which decomposed individual predictions into feature-level contributions. In a representative case with a favorable outcome, the positive contributions of low procalcitonin, high PNI, and low WFNS grade outweighed the negative impact of moderate inflammatory markers. These case-level plots provide a transparent view of how specific variables shaped predictions. LIME analysis was also applied to complement SHAP (Additional file 7: Figure S6). In the representative patient, LIME predicted an 89% probability of a favorable outcome and 11% of an unfavorable outcome. The feature weights showed that higher PNI was strongly associated with a favorable classification, while lower modified Fisher grade and PLR contributed modestly.

### Implementation of the web calculator

**Fig. 8:**
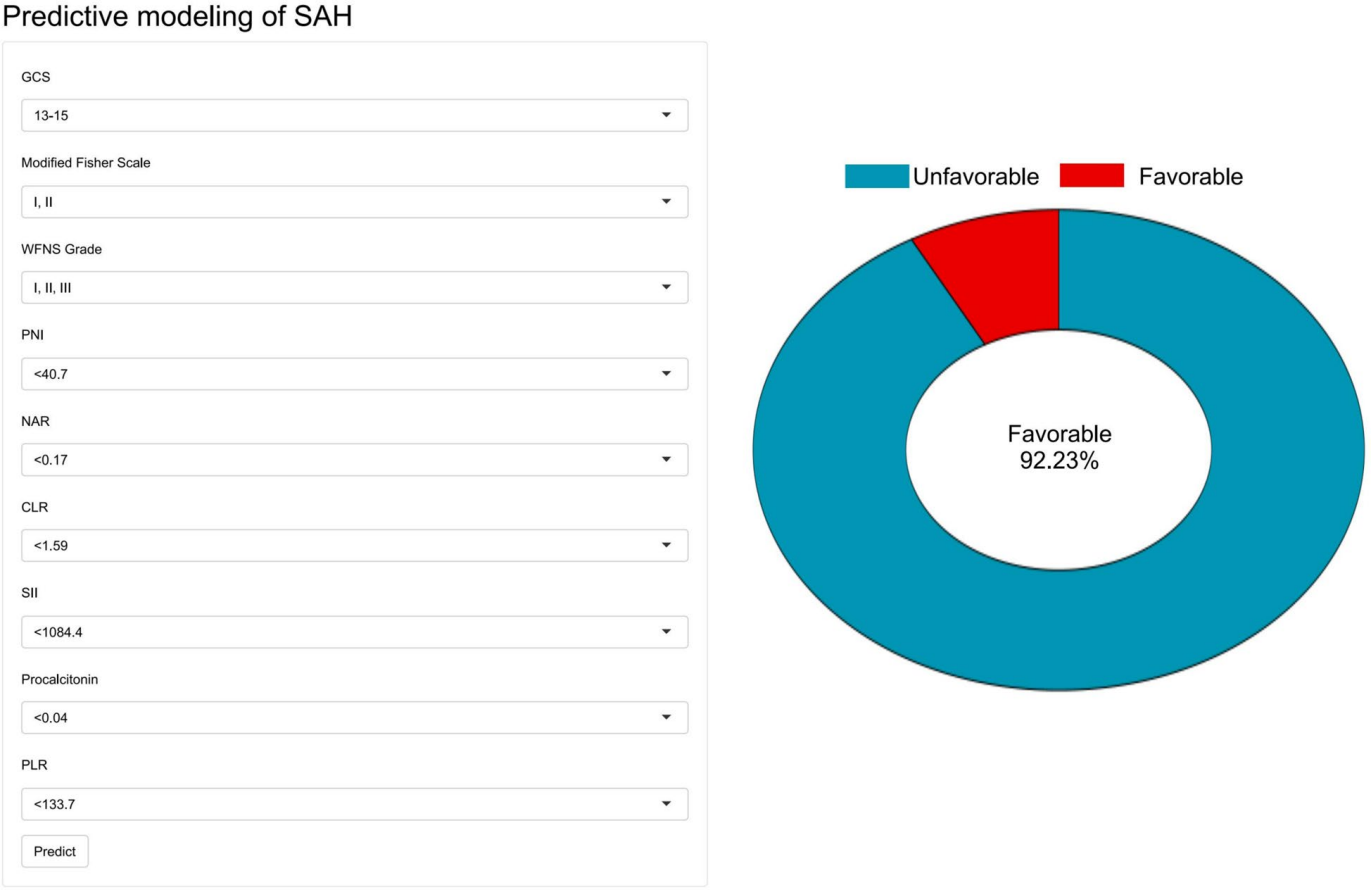
A web-based calculator for predicting short-term prognosis in aSAH patients

The final GBM model was deployed as an interactive web-based risk prediction tool Fig. 8. The interface requires entry of nine variables: GCS score, modified Fisher grade, WFNS grade, PNI, NAR, CLR, SII, procalcitonin, and PLR. After submission, the tool provides an individualized prediction of short-term functional outcome at discharge, accompanied by graphical explanations based on the model’s logic. A web-based application (https://ych-neuro-cqmu2024.shinyapps.io/shinyapp/) was developed to make these predictive models accessible online, utilizing the R package “shiny” for its development [22].

This tool allows early risk stratification at the bedside and may support tailoring of management strategies. It also provides real-time interaction, remote access, and could be integrated into future clinical decision support systems.

## Summary of key findings

Using multicenter real-world data, we developed and validated a GBM model to predict short-term functional outcomes in patients with aSAH. The model incorporated nine clinical and inflammatory variables and showed good discrimination, calibration, and clinical usefulness in both internal and quasi-external cohorts. SHAP analysis demonstrated interpretability of the model and identified SII, procalcitonin, and WFNS grade as major contributors. Finally, an interactive web-based calculator was created to support individualized risk assessment and facilitate clinical use.

## Discussion

This multicenter study leveraged real-world EMR data from four tertiary hospitals to evaluate the predictive performance of six commonly applied machine learning algorithms for short-term functional outcomes in patients with aSAH. Among them, the GBM model showed stable performance across discrimination, calibration, and clinical usefulness, and was selected as the final model. It was further validated in a quasi-external cohort to confirm robustness and generalizability. Unlike previous studies that mainly focused on long-term prognostication (3 to 6 months postoperatively), our model addressed the early “admission to discharge” period, providing early risk stratification during the acute phase of care [23–25]. The model integrated inflammation- and nutrition-related biomarkers and used a two-step feature selection process (Boruta followed by LASSO), which improved stability and interpretability of predictors [26–29]. To improve interpretability, SHAP analysis was applied, and an interactive web-based calculator was created to support clinical application at the bedside.

Previous prognostic models for aSAH have often been developed using public databases such as Dryad and GEO [24, 25], which provide limited data granularity and may not adequately represent Chinese patient populations. Many of these studies relied on logistic regression, which is constrained in capturing complex nonlinear interactions among predictors [23]. By contrast, our study used multicenter real-world data reflecting broader patient characteristics and treatment practices. In addition to establishing training and internal testing cohorts, a quasi-external validation cohort was included to comprehensively evaluate model performance using AUC, Brier score, and decision curve analysis. The GBM algorithm, based on iterative residual optimization and ensemble learning, proved suitable for modeling high-dimensional clinical data, consistent with its reported application in stroke, oncology, and critical illness [30–33]. Continuous predictors were further discretized into quartile-based categories, a strategy that reduced the effect of outliers and improved interpretability for bedside use [34–36].

The outcome distribution in this cohort was moderately imbalanced, with favorable outcomes accounting for about 70%. To address this imbalance, SMOTE was applied during training, which preserved model discrimination and calibration while balancing outcome classes [37]. The final model incorporated nine variables covering three main clinical domains: neurological status, nutritional condition, and systemic inflammation. These variables are routinely available and provide clear clinical interpretability [38–43]. Specifically, the GCS, WFNS grade, and modified Fisher grade capture neurological impairment, hemorrhagic burden, and overall disease severity, and have been widely validated as prognostic markers in aSAH. The inclusion of PNI, which combines serum albumin and lymphocyte count, further highlights the contribution of nutritional and immune status to neurocritical outcomes. Higher PNI has been associated with better prognosis in stroke, cancer, and intensive care settings [44–48], a trend also observed in our study, where patients with favorable outcomes had higher PNI levels. The prognostic value of PNI may be related to its roles in osmotic regulation, antioxidant defense, and immune modulation [44]. Comparative analyses further indicated that integrating traditional grading scales with laboratory biomarkers achieved superior discrimination compared with either domain alone, supporting the added value of combining clinical and biological data [49].

Inflammatory markers contributed substantially to model performance, underscoring the role of systemic inflammation as a secondary injury mechanism in aSAH. NAR, CLR, SII, PLR, and procalcitonin showed consistently high importance across models, supporting their robustness as predictors. NAR and SII act as integrated indices of inflammatory activity and immune balance, and are commonly used in the risk assessment of stroke and cardiovascular diseases [50–56]. CLR reflects the balance between immunosuppression and acute inflammation [57, 58]. PLR captures the link between coagulation and inflammatory responses and has been associated with complications such as delayed cerebral ischemia [59]. Elevated procalcitonin, in addition to indicating infectious complications, may also reflect systemic inflammatory dysregulation and has been linked to worse neurological outcomes in previous studies [60, 61]. Our findings indicate that inflammatory markers are useful not only as predictive features but also as possible markers for monitoring disease progression in aSAH. Although several inflammation-based indices are mathematically correlated, variance inflation factor analysis showed that all selected predictors had VIFs < 5, indicating acceptable levels of collinearity [62, 63].

Given the limitations of “black-box” machine learning models in clinical practice, SHAP analysis was applied to clarify both global and individual-level contributions of predictors within the GBM model. Summary plots showed that SII, procalcitonin, and WFNS grade were among the most influential features. Waterfall and force plots provided case-level explanations of how individual predictors shaped outcome estimates. These visualization methods may improve understanding of model predictions and support their clinical use. To complement SHAP, LIME was also applied to generate case-level explanations, which addressed some limitations of SHAP in correlated settings and further improved interpretability [64]. To enhance clinical applicability, we further developed a publicly accessible online risk calculator, allowing rapid entry of the nine selected variables and immediate output of individualized risk estimates, which may assist decision-making at the bedside.

Despite its strengths (multicenter design, algorithmic comparison, external validation, and emphasis on interpretability), this study has several limitations. First, as a retrospective study, it is subject to selection bias and unmeasured confounding, which may affect causal inference. Second, all inflammatory markers were measured at a single time point; future models may benefit from incorporating longitudinal or time-series data to capture dynamic changes. Third, imaging-based indicators such as the modified Fisher grade rely on manual assessment and may be influenced by interobserver variability; integration of automated image analysis could improve objectivity and consistency. Fourth, due to inter-institutional differences in data recording, some clinically relevant variables such as postoperative complications and vasospasm were not included, which may have limited the scope of the model. Fifth, subgroup and sensitivity analyses were not conducted, which restricts evaluation of model robustness in specific patient subsets or under alternative assumptions. Sixth, although quasi-external validation was performed, the cohort came from a single institution within the same healthcare system, which may limit generalizability [65]. Finally, although a web-based calculator was developed, its clinical impact and value for guiding interventions have not yet been tested in prospective studies and warrant further investigation.

## Conclusions

In this study, we developed a machine learning model using EMR data from three hospitals for training and validated it in an additional quasi-external cohort from a fourth hospital to predict short-term outcomes in patients with aneurysmal subarachnoid hemorrhage (aSAH). The model showed good predictive accuracy and calibration. Our analysis highlighted the prognostic importance of clinical and laboratory indicators, including GCS score, modified Fisher grade, WFNS grade, PNI, NAR, CLR, SII, procalcitonin, and PLR, which support early risk stratification and may help guide rehabilitation planning after discharge. These findings suggest that incorporating routinely available clinical and inflammatory markers could contribute to more individualized care. Future studies should aim to enlarge cohort size, validate the model in diverse clinical settings, and integrate dynamic variables and longitudinal follow-up to improve clinical applicability and generalizability.

## Supplementary Information

Additional file 1. Table S1: Collinearity Analysis of Candidate and Selected Variables.

Additional file 2. Fig S1: Comparison of feature selection before and after quartile-based binning. (A-C) Results of Boruta combined with LASSO before binning; (D-F) results after binning. The figure contrasts variable importance ranking and feature retention under the two preprocessing strategies.

Additional file 3. Fig S2: Ten-fold cross-validation ROC curves in the training set. ROC curves of six machine learning models under 10-fold cross-validation: (A) RF, (B) GBM, (C) SVM, (D) LR, (E) XGBoost, and (F) NN. Each curve corresponds to one fold; the mean ROC and 95% CI are also presented, and AUC values with 95% CIs are reported.

Additional file 4. Fig S3: Calibration curves of six machine learning models. (A) Training set; (B) test set. Calibration curves for LR, RF, GBM, ANN, SVM, and XGBoost. The x-axis represents predicted probability, and the y-axis denotes observed incidence. The dashed line indicates perfect calibration, while colored lines reflect actual model performance.

Additional file 5. Fig S4: Model performance after SMOTE oversampling. (A-C) ROC, calibration, and decision curve analyses in the training set; (D-F) corresponding results in the test set.

Additional file 6. Fig S5: Comparison of model performance and DeLong tests. ROC curves of three models (ModA, ModB, ModC) are shown in panel A, and decision curve analysis in panel B compares net clinical benefit across probability thresholds against treat-all and treat-none strategies. ModA incorporated all nine predictors (GCS score, modified Fisher grade, WFNS grade, PNI, NAR, CLR, SII, procalcitonin, and PLR). ModB included only traditional clinical scores (GCS score, modified Fisher grade, and WFNS grade). ModC was restricted to inflammation- and nutrition-related markers (PNI, NAR, CLR, SII, procalcitonin, and PLR).

Additional file 7. Fig S6: LIME interpretation of the GBM model. (A) Ranked feature contributions: bar length indicates contribution magnitude, and colors denote direction of effect. The top nine predictors were CLR, WFNS, PNI, GCS, PLR, modified Fisher grade, procalcitonin, NAR, and SII. (B) Example case prediction: probability of favorable outcome 0.89 and unfavorable outcome 0.11. WFNS ≤ 1 and higher GCS contributed positively, whereas elevated CLR had a negative impact.Note:PNI 1 (<40.7),2 (40.7,45.0),3 (45.0,48.8),4 (>48.8), NAR 1 (<0.17),2 (0.17,0.24),3 (0.24,0.31),4 (>0.31), PLR 1 (<133.7),2 (133.7,208),3(208,295),4(>295),SII 1 (<1084.4),2 (1084.4,1860.8),3 (1860.8,3037.3),4 (>3037.3),SIRI 1 (<2.57),2 (2.57,5.08),3 (5.08,9.68),4(>9.68), Procalcitonin 1 (<0.04),2

## Data Availability

The datasets analyzed during the current study are available from the corresponding author on reasonable request. The source code and deployment files for the web-based calculator are publicly available on GitHub at https://github.com/jaychou0514/R-ych-neuro-cqmu2024. The predictive model developed in this study has been successfully deployed on a web platform for further verification and use by readers (https://ych-neuro-cqmu2024.shinyapp.io/shinyapp/).

## Abbreviations

Asah: Aneurysmal subarachnoid hemorrhage
EMR: Electronic medical records
LASSO: Least Absolute Shrinkage and Selection Operator
RF: Random forest
SVM: Support vector machine
GBM: Gradient boosting machine
XGBoost: Extreme gradient boosting
AUC: Area under the curve
DCA: Decision curve analysis
WFNS: World Federation of Neurosurgical Societies
DCI: Delayed cerebral ischemia
CVS: Cerebral vasospasm
NLR: Neutrophil-to-lymphocyte ratio
PLR: Platelet-to-lymphocyte ratio
SII: Systemic immune-inflammation index
PNI: Prognostic nutritional index
CLR: C-reactive protein-to-lymphocyte ratio
ML: Machine learning
SHAP: Shapley Additive Explanations
STROBE: The Strengthening the Reporting of Observational Studies in Epidemiology
CTA: Computed tomography angiography
DSA: Digital subtraction angiography
GCS: Glasgow Coma Scale
CRP: C-reactive protein
NLPR: Neutrophil-lymphocyte-platelet ratio
MLR: Monocyte-to-lymphocyte ratio
SIRI: Systemic inflammation response index
AISI: Aggregate index of systemic inflammation
mRS: Modified Rankin Scale
IQRs: Interquartile ranges
ROC: Receiver operating characteristic
PAR: Platelet-to-Albumin Ratio
CI: Confidence interval
SMOTE: Synthetic minority oversampling technique
VIF: Variance inflation factor
