# Supplementary material for: Machine learning-based prediction of short-term outcomes in aneurysmal subarachnoid hemorrhage: a multicenter study integrating clinical and inflammatory indicators
Source: BMC Med. 2025 Nov 29;24:7. doi: 10.1186/s12916-025-04523-y (PMC12772089; doi:10.1186/s12916-025-04523-y)
Supplement: Supplementary file 1 — Additional file 1. Table S1: Collinearity Analysis of Candidate and Selected Variables. [file 12916_2025_4523_MOESM1_ESM.zip › Additional file 1/Table.S1.docx]

Supplementary Table 1: Collinearity Analysis of Candidate and Selected Variables.

| **Variable** | **VIF (All candidate variables)** | **VIF (Selected features)** | **Inclusion in final model** |
| --- | --- | --- | --- |
| Sex | 1.402935755256102 |  |  |
| Age | 1.235897805115142 |  |  |
| Hypertension | 1.159750839349026 |  |  |
| Diabets | 1.11565893499491 |  |  |
| **GCS** | 5.104540209895452 | 4.088641300493407 | Yes |
| **Modified fisher** | 1.500976373462231 | 1.252243155811892 | Yes |
| **Wfns** | 4.884543232090635 | 3.739443038179911 | Yes |
| Hunthess | 2.220408744292216 |  |  |
| Ddimer | 1.208334765614061 |  |  |
| Surgical method | 1.183484824130964 |  |  |
| Albumin | 2.06267720786592 |  |  |
| Fibrinogen | 1.2001024263612 |  |  |
| Aneurysm location | 1.151824718400607 |  |  |
| **PNI** | 1.934654650638077 | 1.176344001395496 | Yes |
| **NAR** | 1.801544778408242 | 1.265140509039768 | Yes |
| PAR | 3.13516467094174 |  |  |
| NLPR | 3.809884026911829 |  |  |
| **PLR** | 2.952536802025027 | 1.534001969453208 | Yes |
| MLR | 3.548729131799127 |  |  |
| **CLR** | 1.452384162981394 | 1.257944272469845 | Yes |
| **SII** | 2.45440119234438 | 1.971383005914274 | Yes |
| AISI | 3.928471073837534 |  |  |
| SIRI | 5.591380815639903 |  |  |
| **Procalcitonin** | 1.265188473208235 | 1.090038023812116 | Yes |
| Sugar | 1.11070207375089 |  |  |
| Creatinine | 1.236488761835307 |  |  |
| RBC | 2.851801313033739 |  |  |
| Hb | 2.783230461137606 |  |  |

**Abbreviations:** GCS = Glasgow Coma Scale; WFNS = World Federation of Neurosurgical Societies; PNI = Prognostic Nutritional Index; NAR = Neutrophil-to-Albumin Ratio; PAR = Platelet-to-Albumin Ratio; NLPR = Neutrophil-to-Lymphocyte-to-Platelet Ratio; PLR = Platelet-to-Lymphocyte Ratio; MLR = Monocyte-to-Lymphocyte Ratio; CLR = C-reactive protein-to-Lymphocyte Ratio; SII = Systemic Immune-Inflammation Index; AISI = Aggregate Index of Systemic Inflammation; SIRI = Systemic Inflammation Response Index.

**Note:** Variance inflation factors (VIFs) were calculated for all candidate variables and for those considered for model inclusion. Only feature variables with VIF < 5 were ultimately selected. ‘Yes’ denotes inclusion in the final model.
