# Supplementary figures and images for: Machine learning-based prediction of short-term outcomes in aneurysmal subarachnoid hemorrhage: a multicenter study integrating clinical and inflammatory indicators
Source: BMC Med. 2025 Nov 29;24:7. doi: 10.1186/s12916-025-04523-y (PMC12772089; doi:10.1186/s12916-025-04523-y)

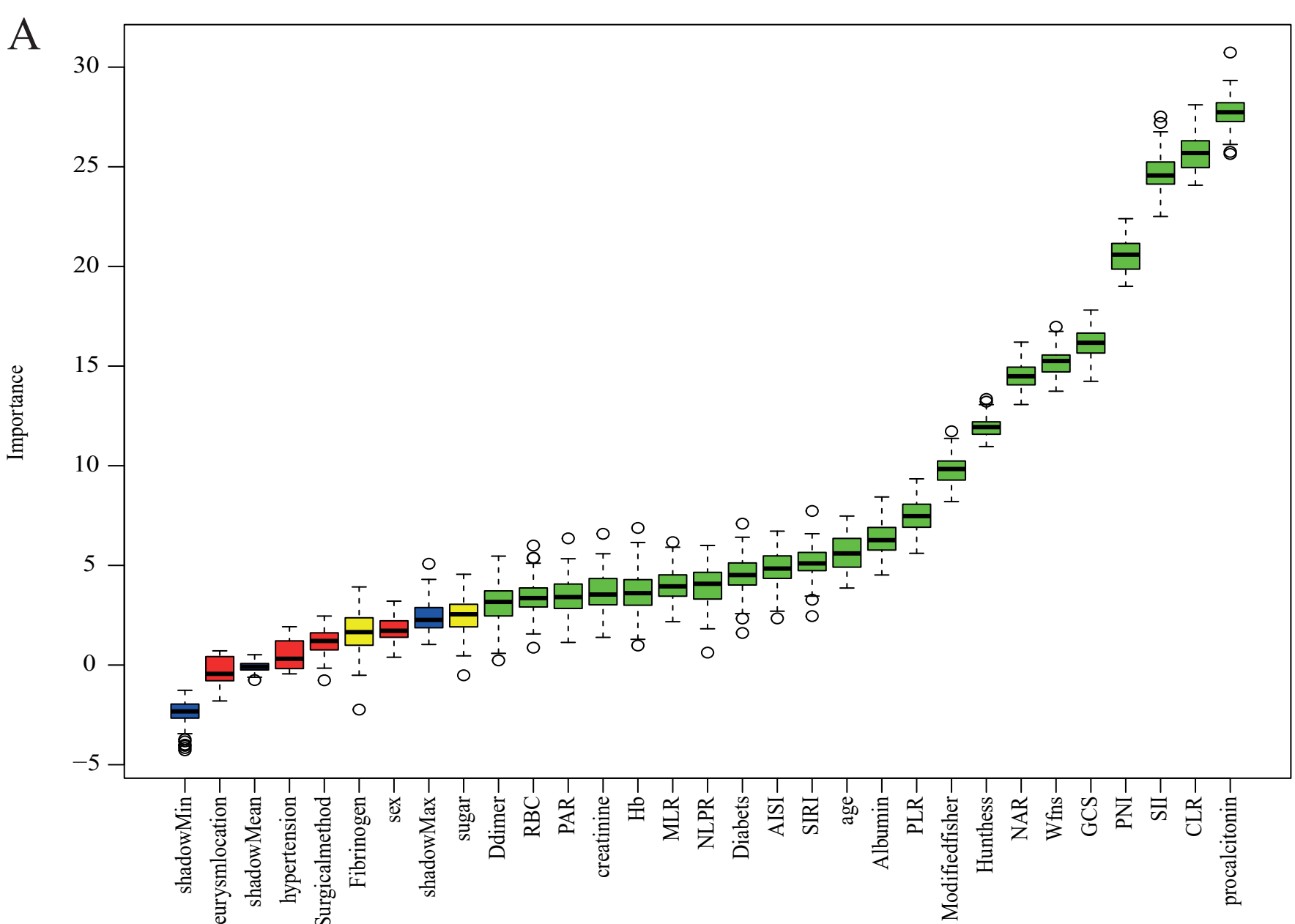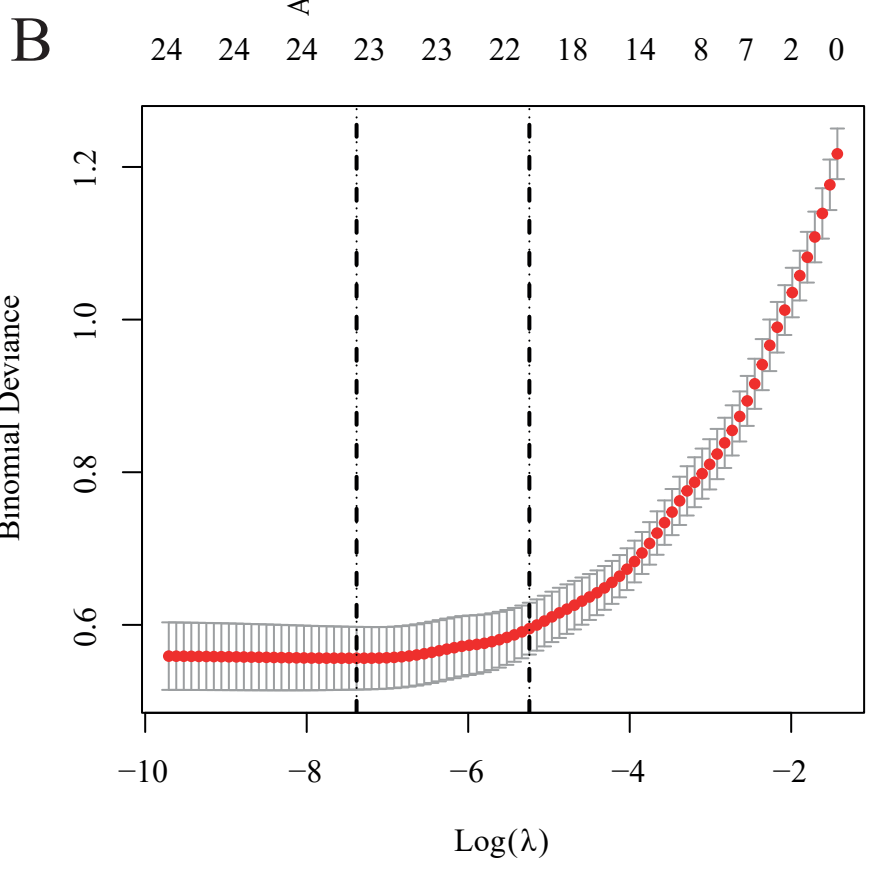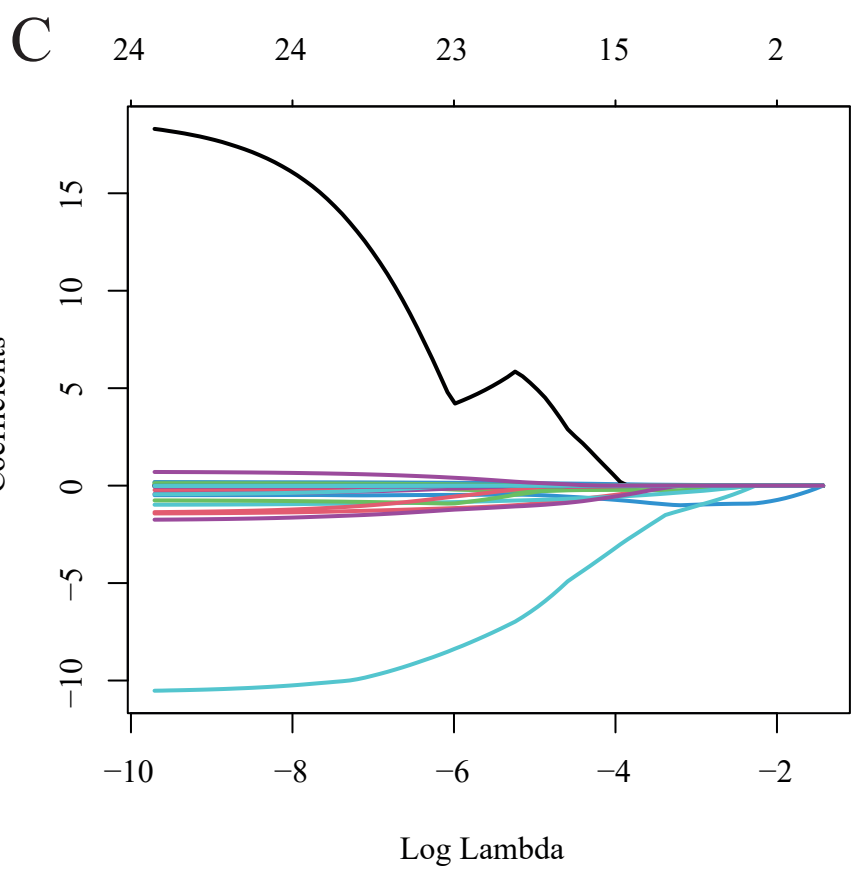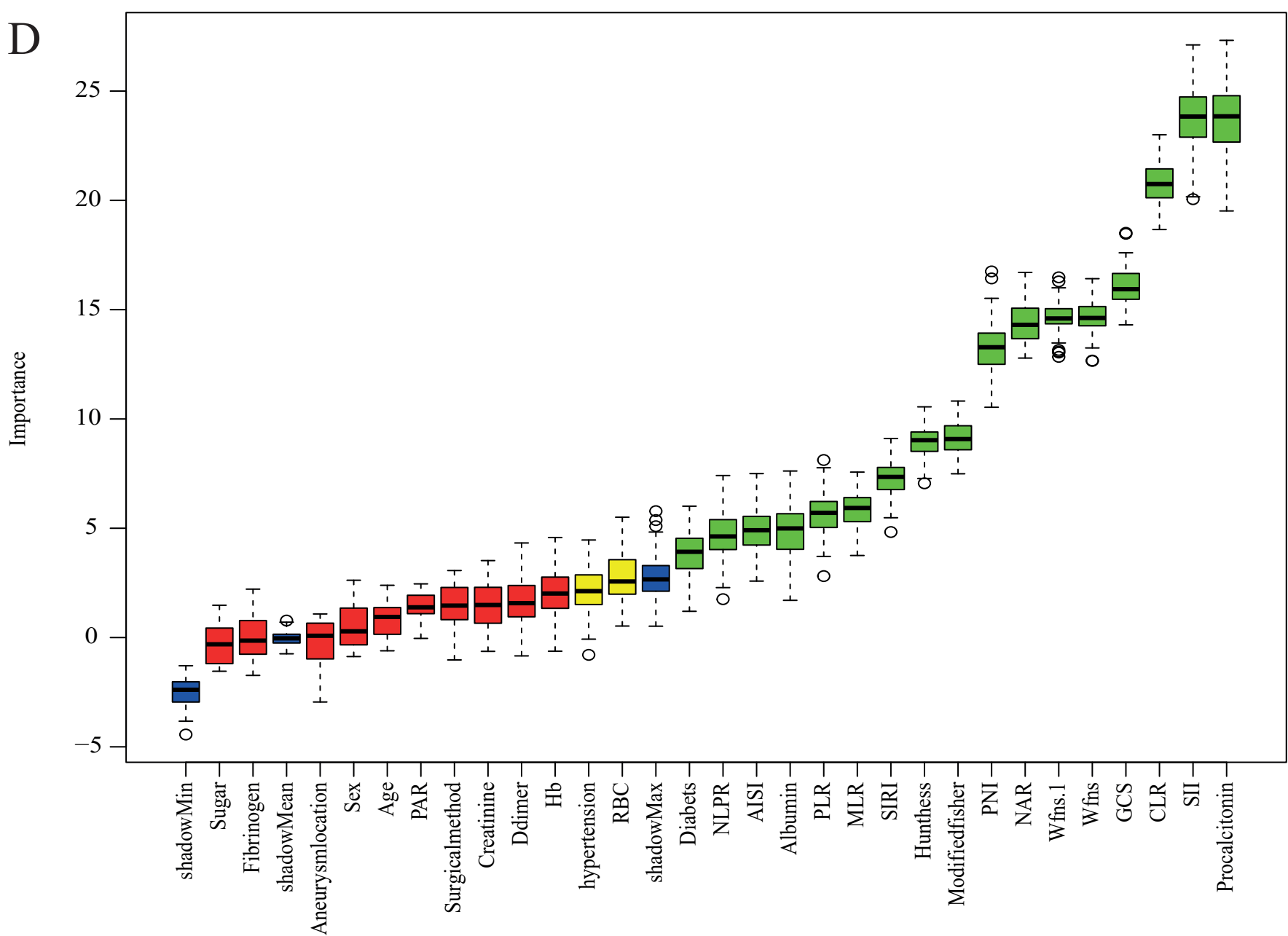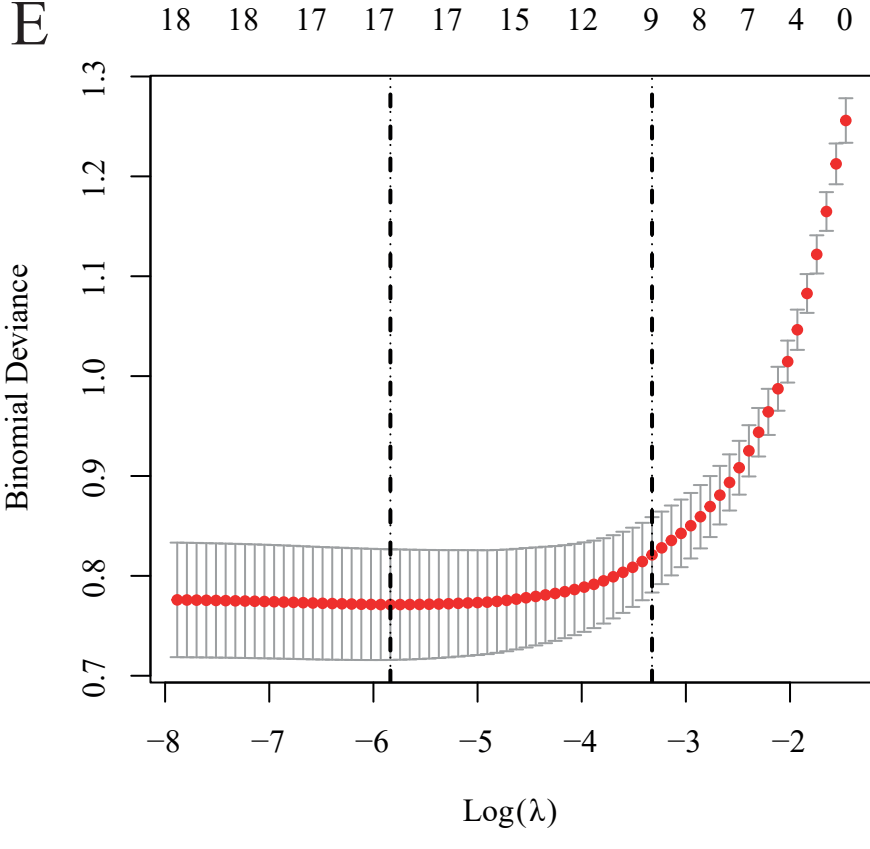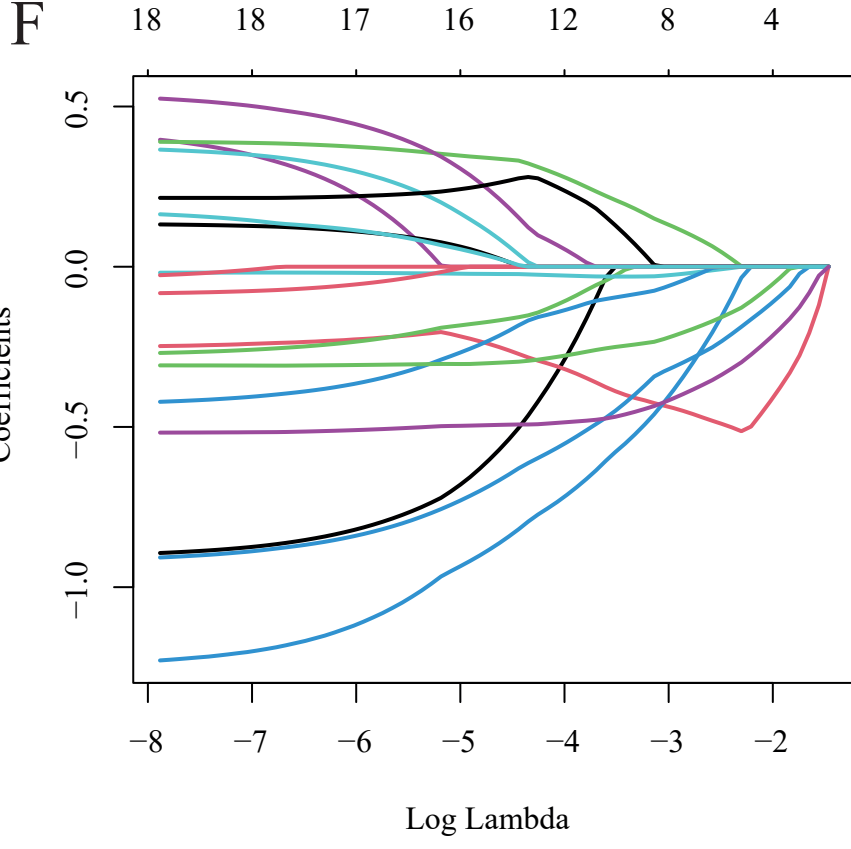

Supplement: Supplementary file 2 — Additional file 2. Fig S1: Comparison of feature selection before and after quartile-based binning. (A-C) Results of Boruta combined with LASSO before binning; (D-F) results after binning. The figure contrasts variable importance ranking and feature retention under the two preprocessing strategies. [file 12916_2025_4523_MOESM2_ESM.zip › Additional file 2/Fig. S1.pdf]

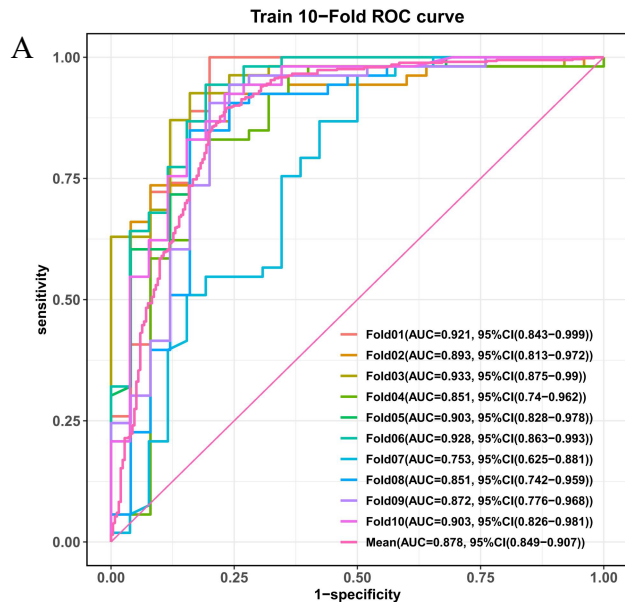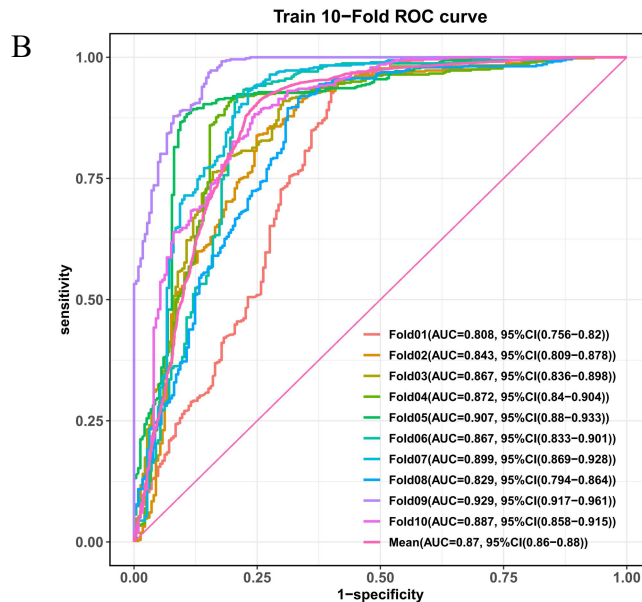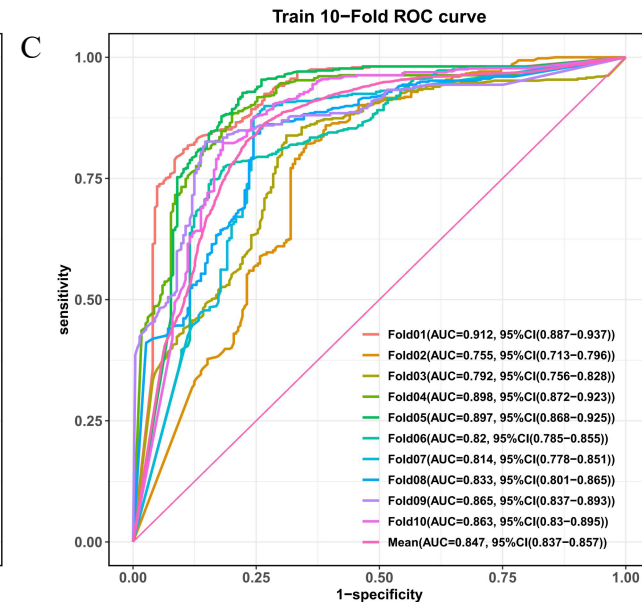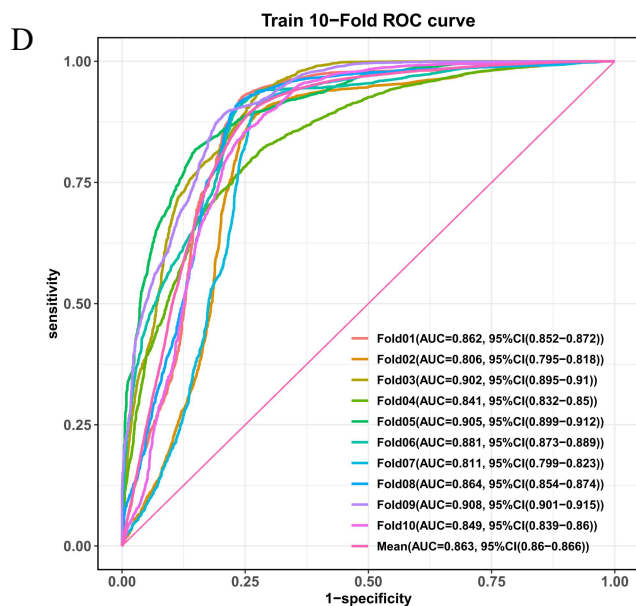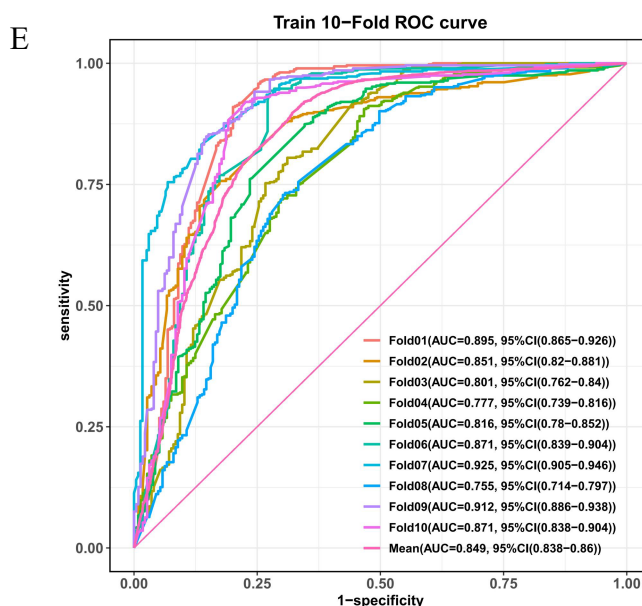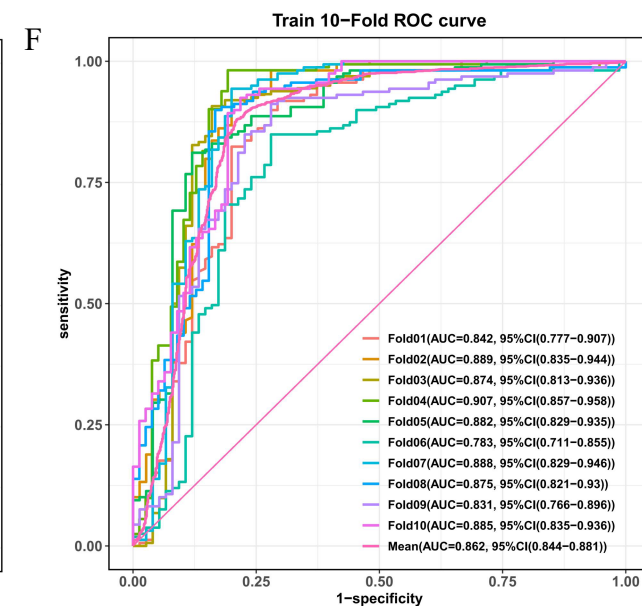

Supplement: Supplementary file 3 — Additional file 3. Fig S2: Ten-fold cross-validation ROC curves in the training set. ROC curves of six machine learning models under 10-fold cross-validation: (A) RF, (B) GBM, (C) SVM, (D) LR, (E) XGBoost, and (F) NN. Each curve corresponds to one fold; the mean ROC and 95% CI are also presented, and AUC values with 95% CIs are reported. [file 12916_2025_4523_MOESM3_ESM.zip › Additional file 3/Fig. S2.pdf]

**A** Train Set: Calibration Curves

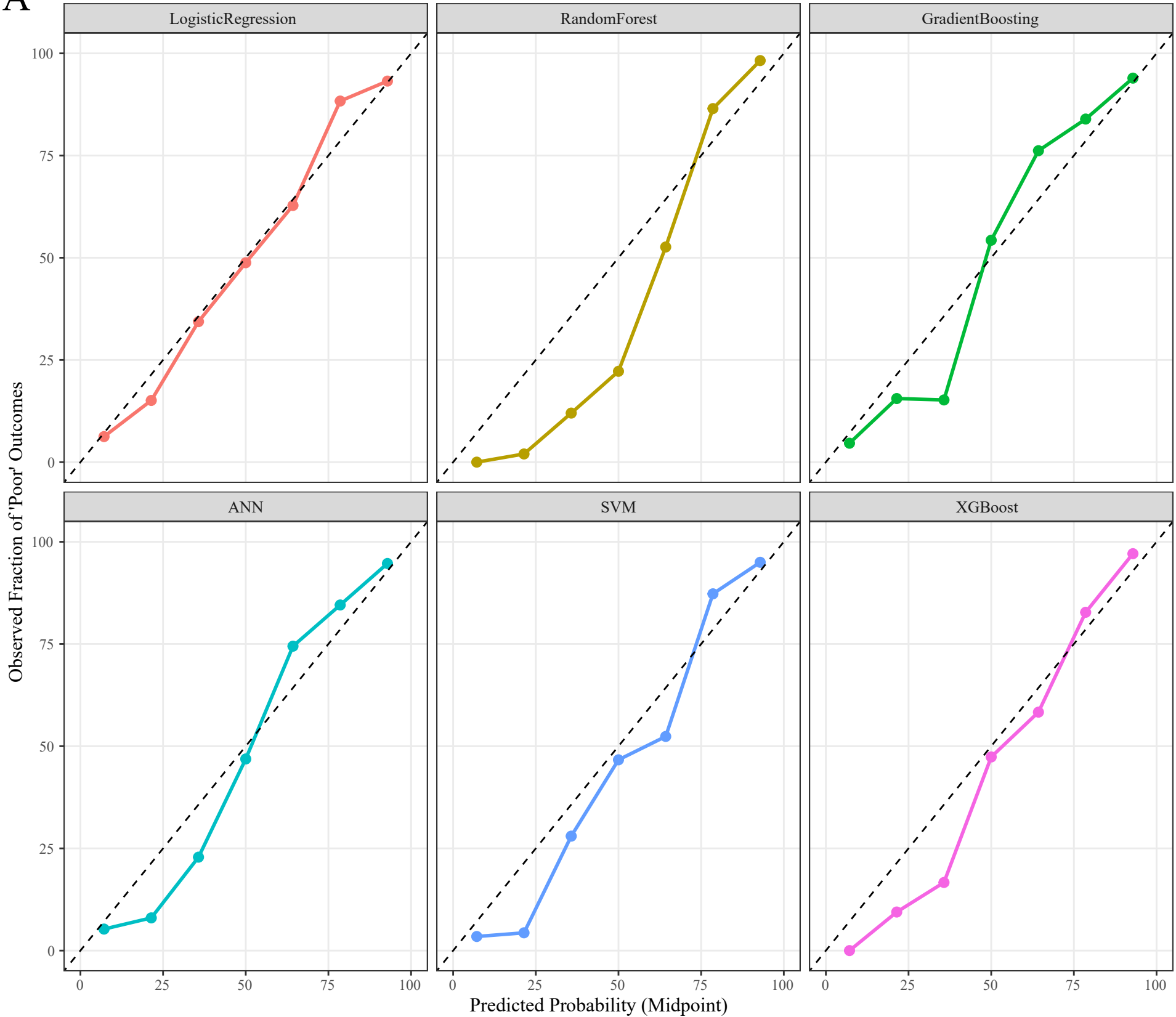

**B** Test Set: Calibration Curves

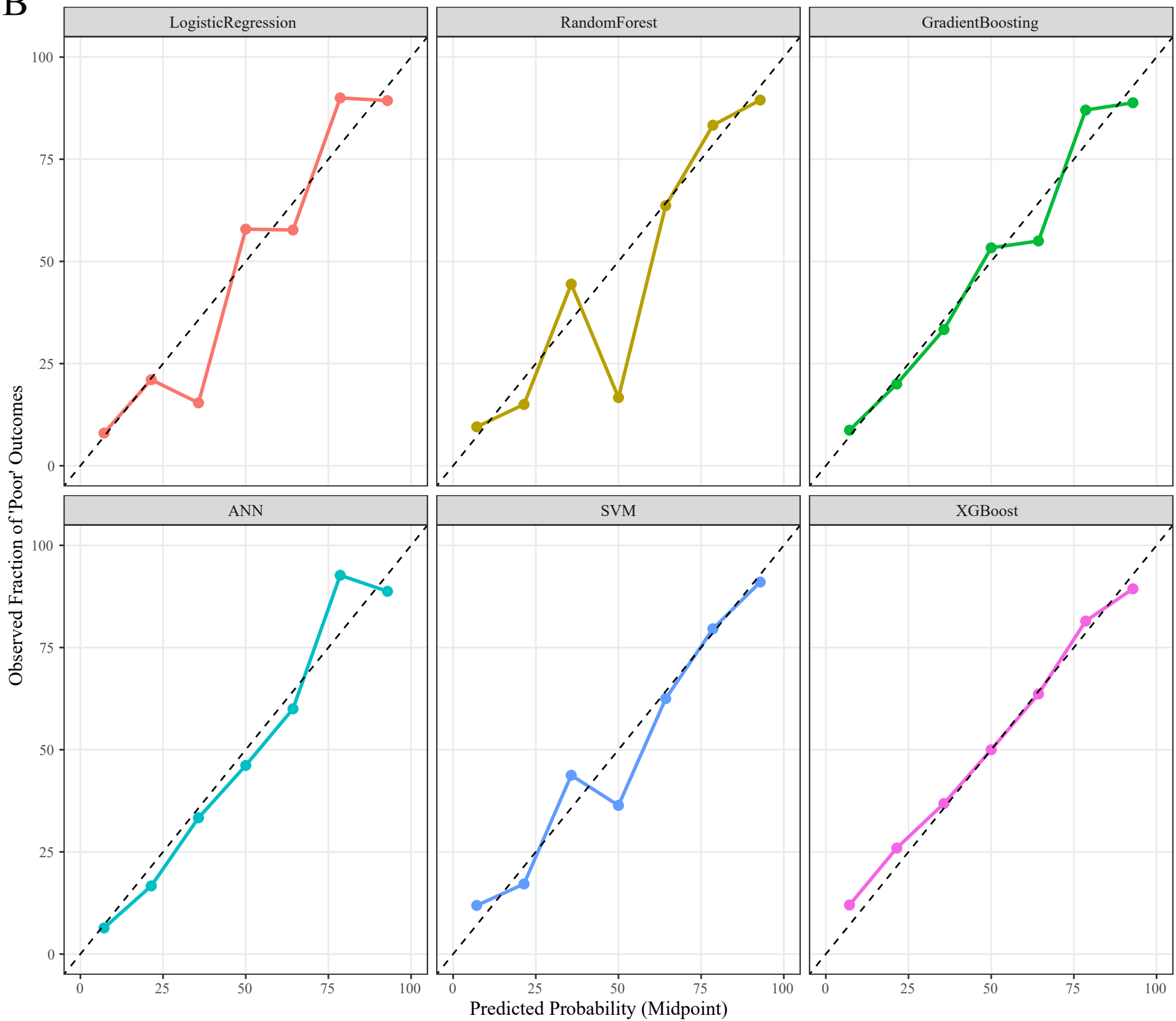

Supplement: Supplementary file 4 — Additional file 4. Fig S3: Calibration curves of six machine learning models. (A) Training set; (B) test set. Calibration curves for LR, RF, GBM, ANN, SVM, and XGBoost. The x-axis represents predicted probability, and the y-axis denotes observed incidence. The dashed line indicates perfect calibration, while colored lines reflect actual model performance. [file 12916_2025_4523_MOESM4_ESM.zip › Additional file 4/Fig. S3.pdf]

A

Local explanation for class Favorable

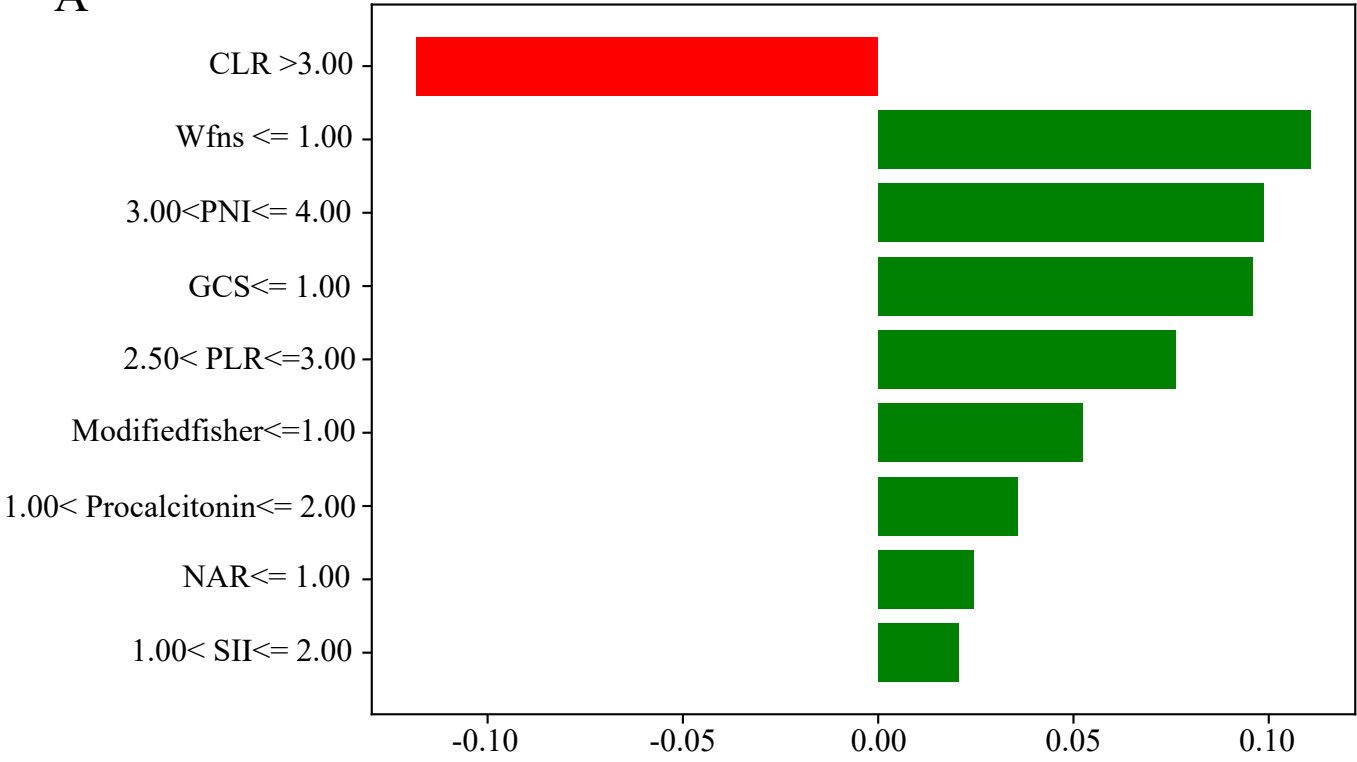

B

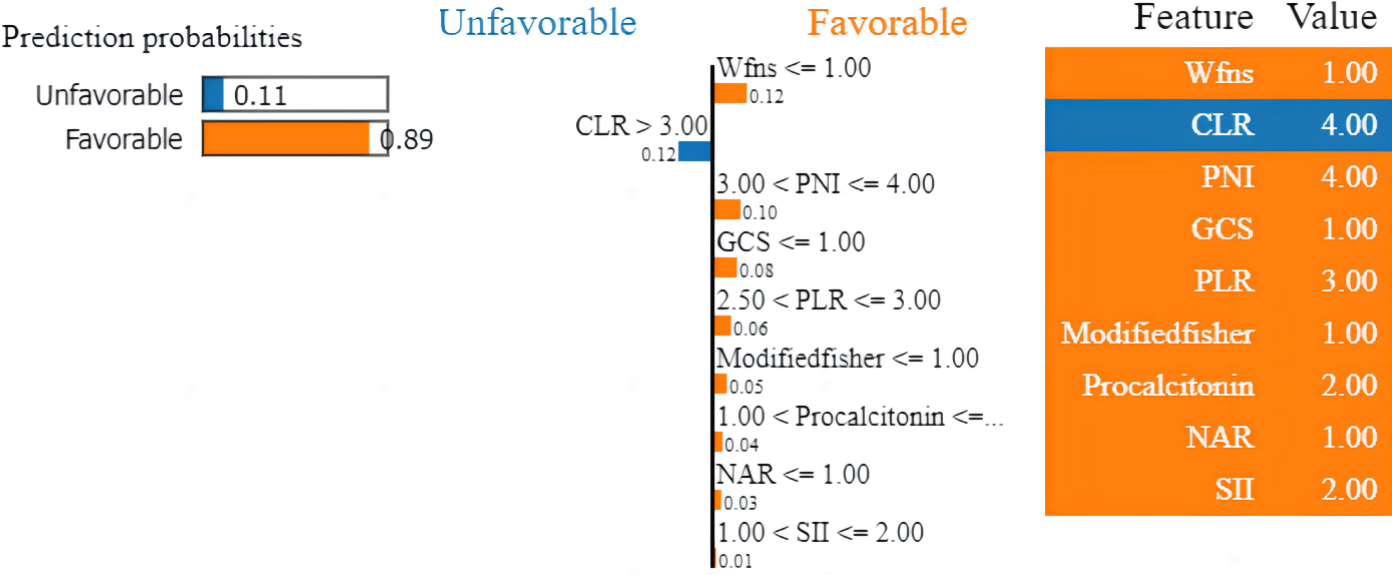

Supplement: Supplementary file 4 — Additional file 4. Fig S3: Calibration curves of six machine learning models. (A) Training set; (B) test set. Calibration curves for LR, RF, GBM, ANN, SVM, and XGBoost. The x-axis represents predicted probability, and the y-axis denotes observed incidence. The dashed line indicates perfect calibration, while colored lines reflect actual model performance. [file 12916_2025_4523_MOESM4_ESM.zip › Additional file 7/Fig. S6.pdf]

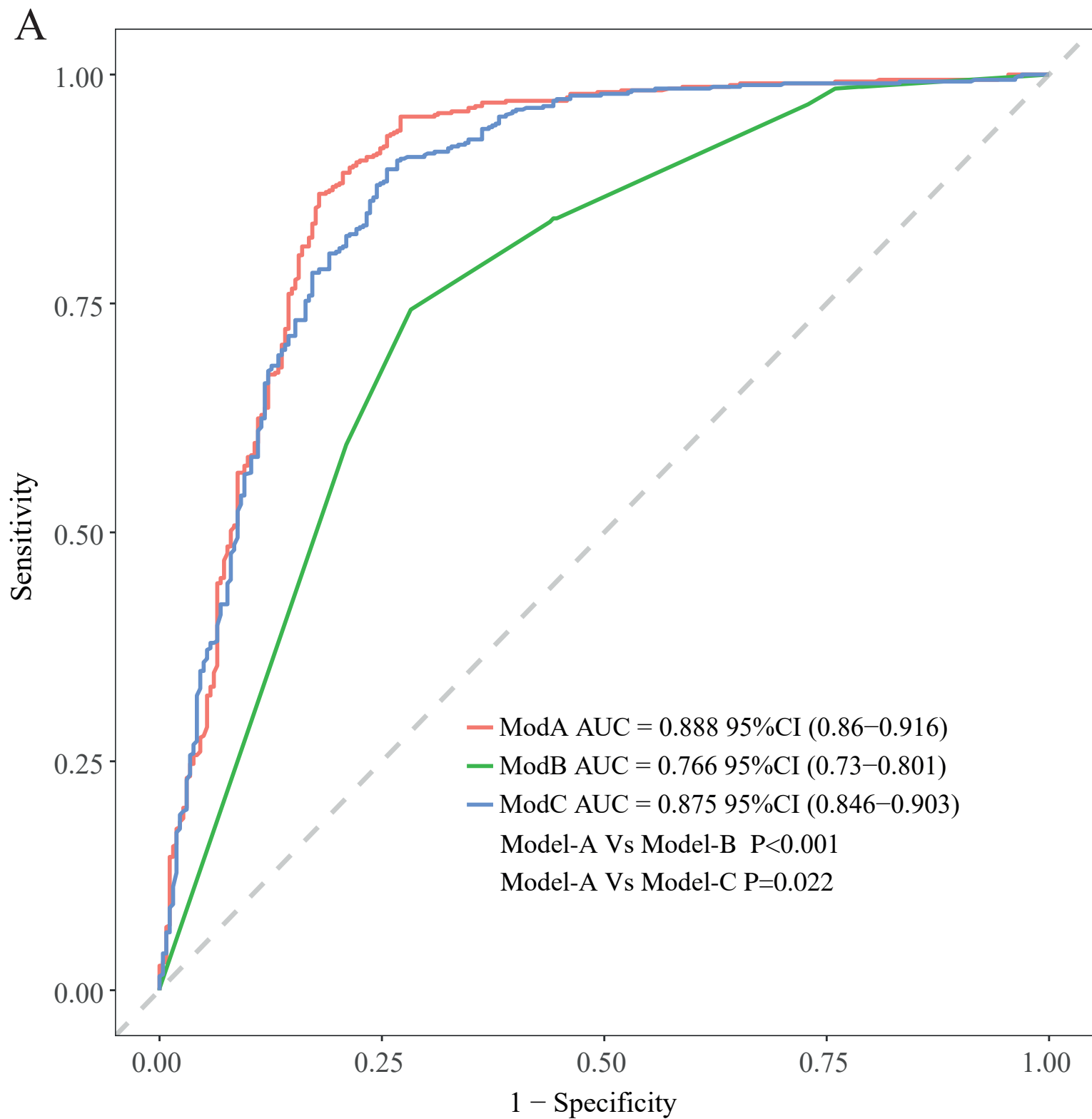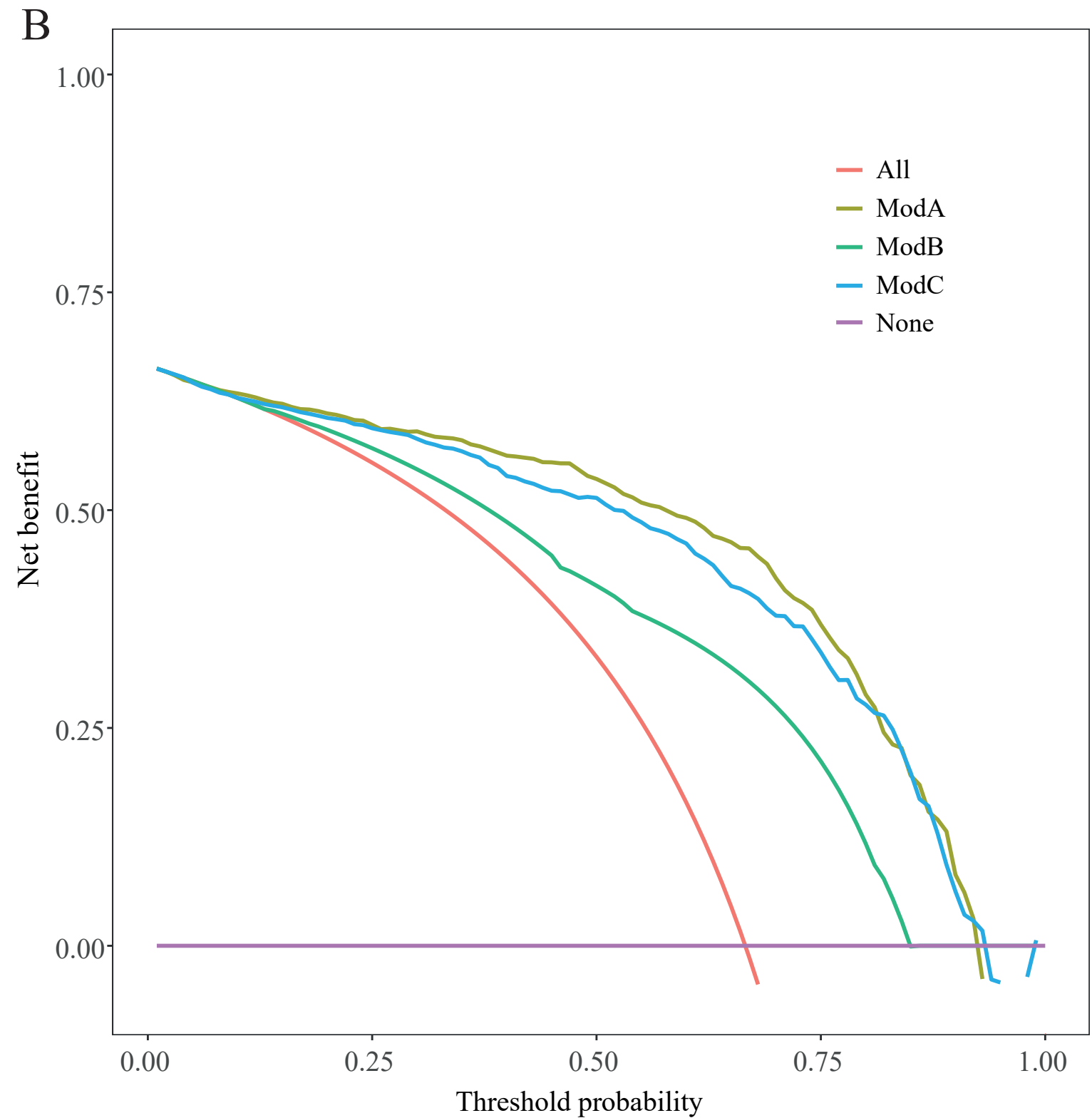

Supplement: Supplementary file 4 — Additional file 4. Fig S3: Calibration curves of six machine learning models. (A) Training set; (B) test set. Calibration curves for LR, RF, GBM, ANN, SVM, and XGBoost. The x-axis represents predicted probability, and the y-axis denotes observed incidence. The dashed line indicates perfect calibration, while colored lines reflect actual model performance. [file 12916_2025_4523_MOESM4_ESM.zip › Additional file 6/Fig. S5.pdf]

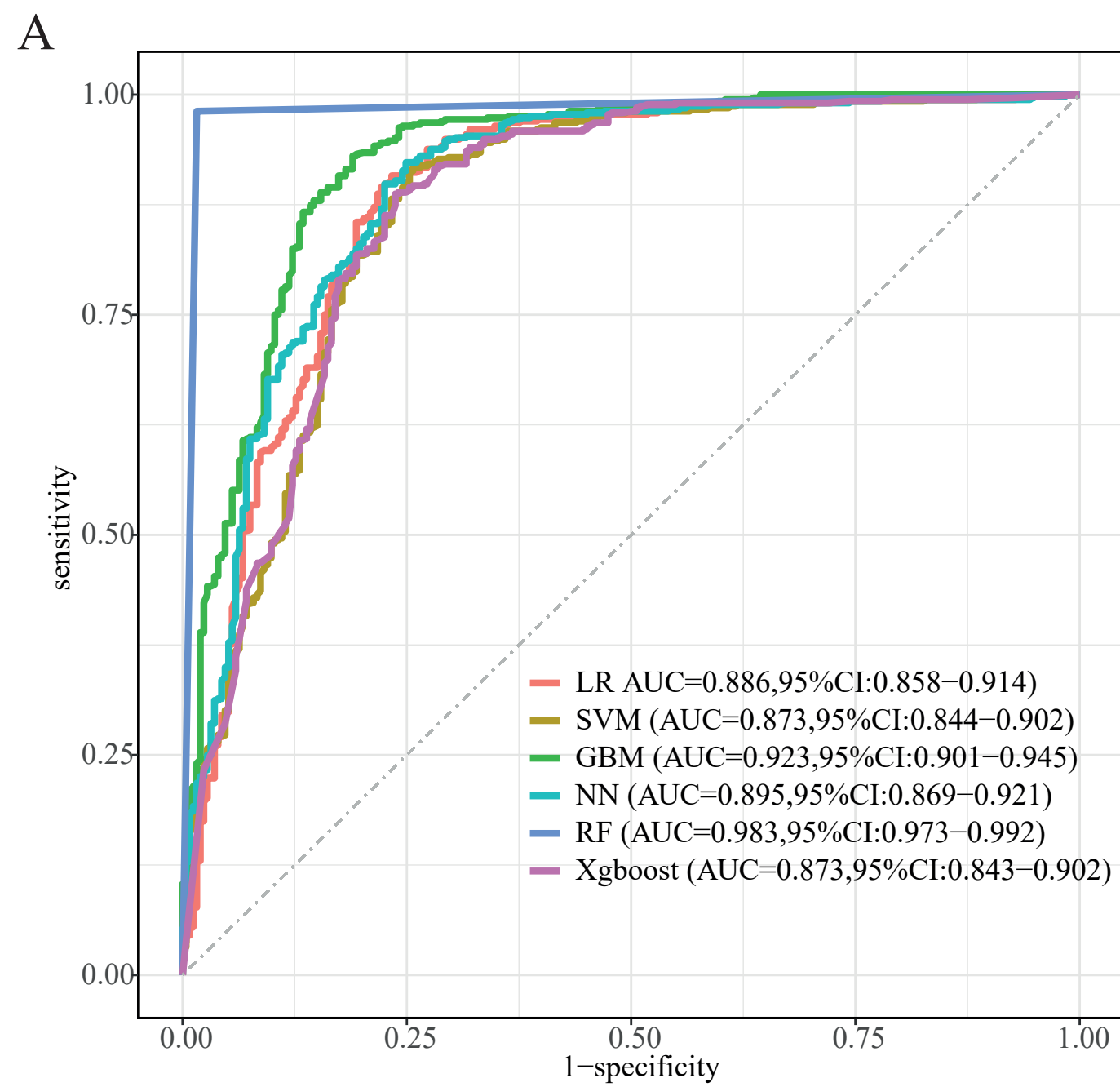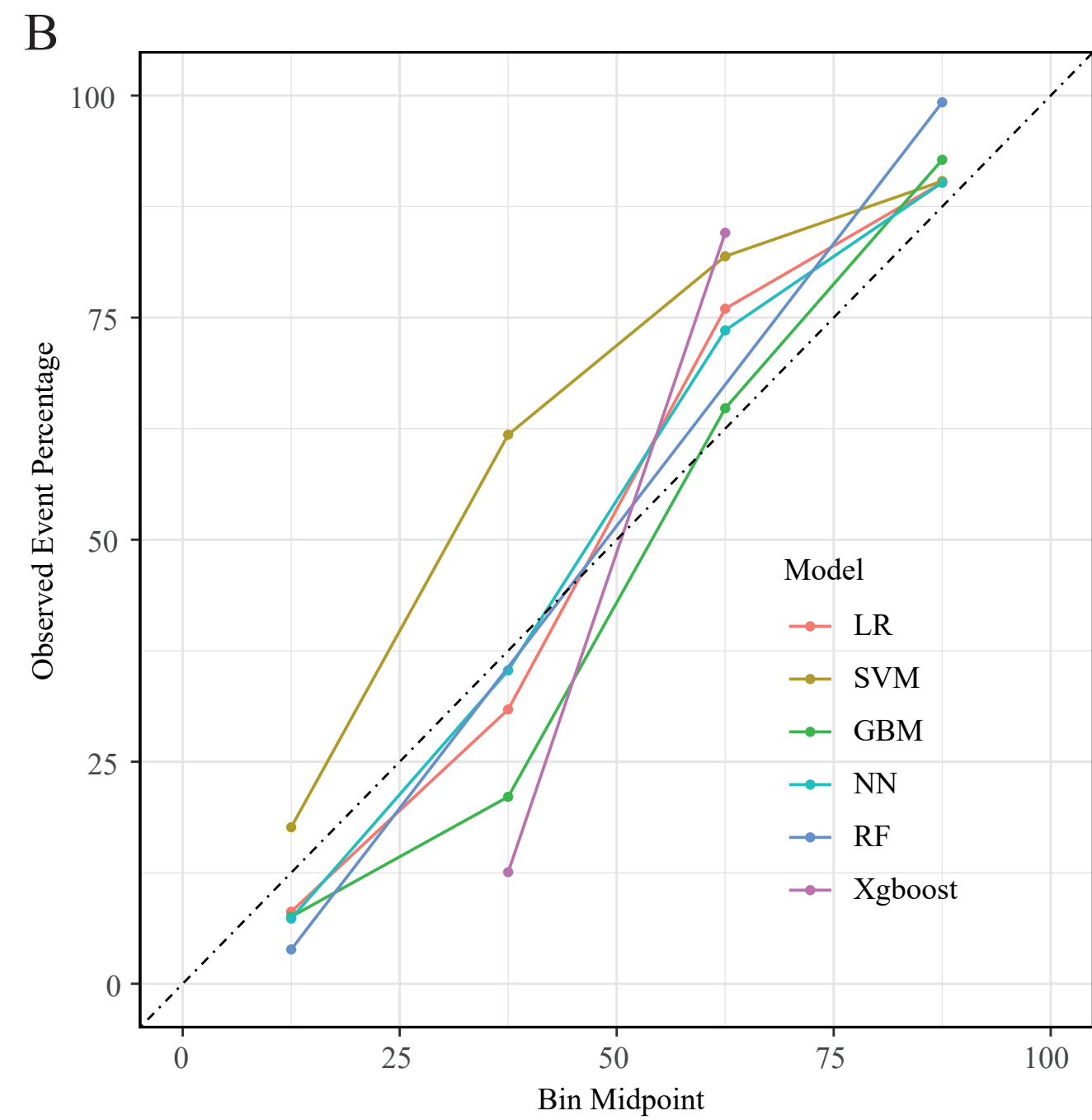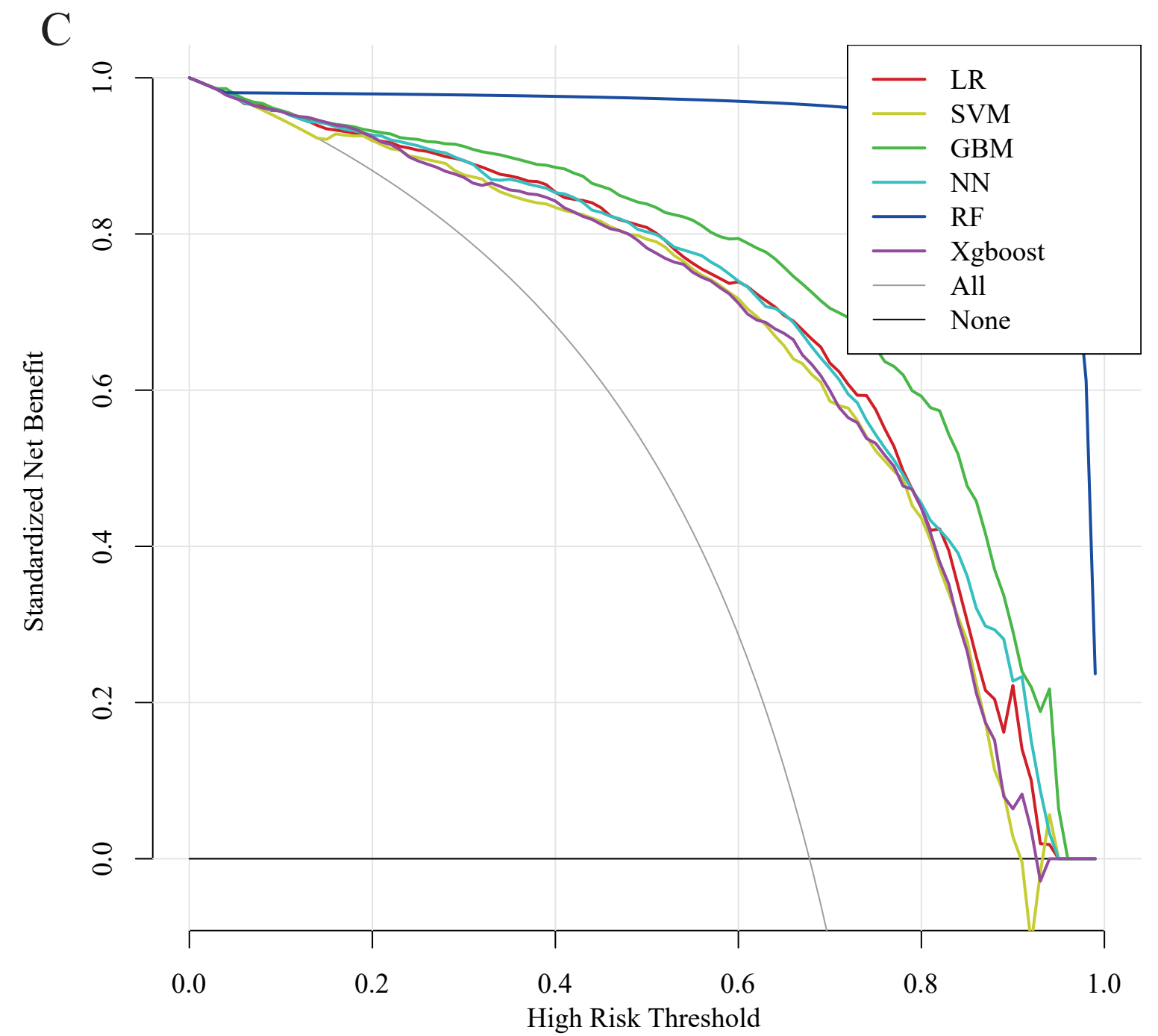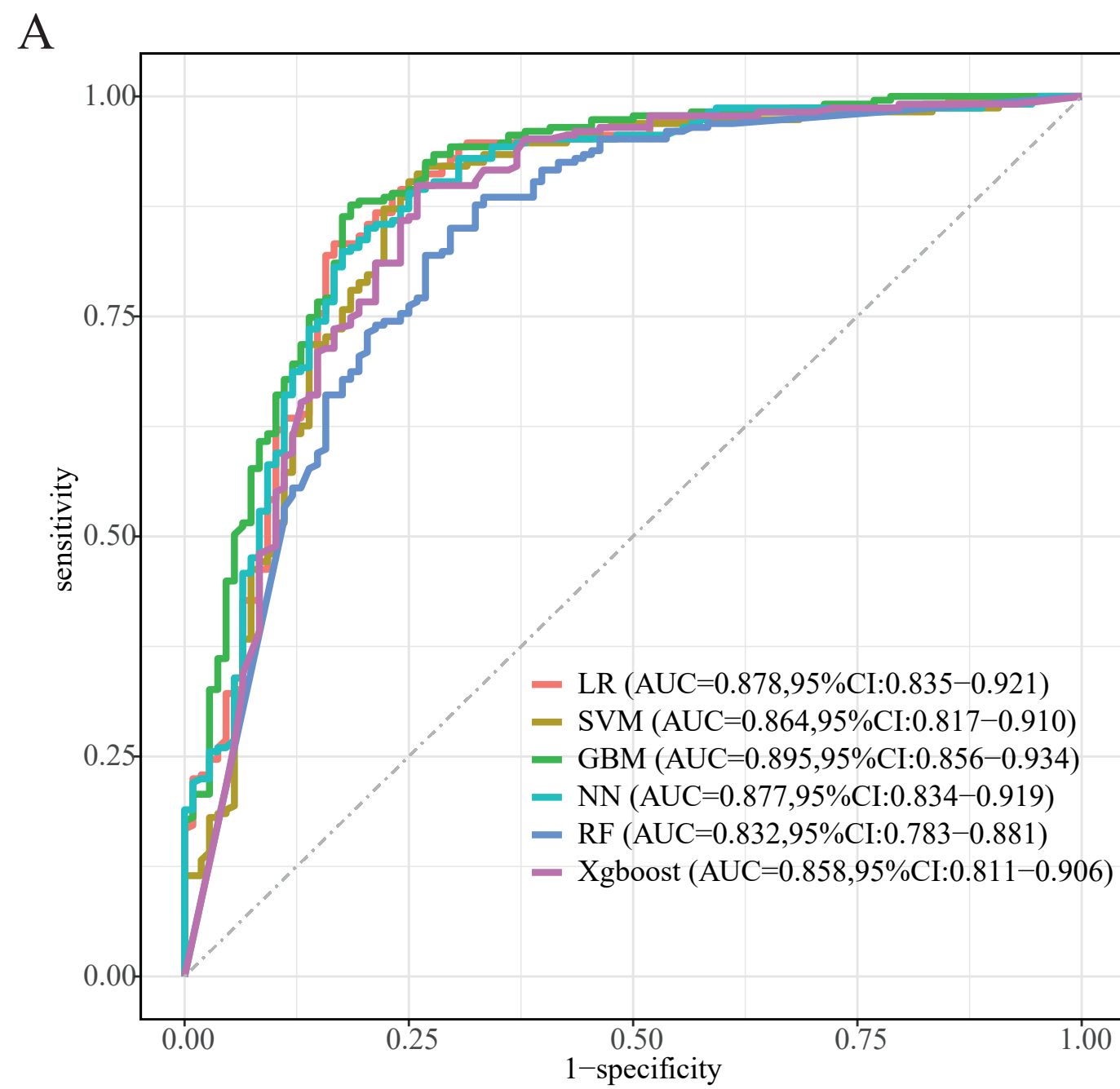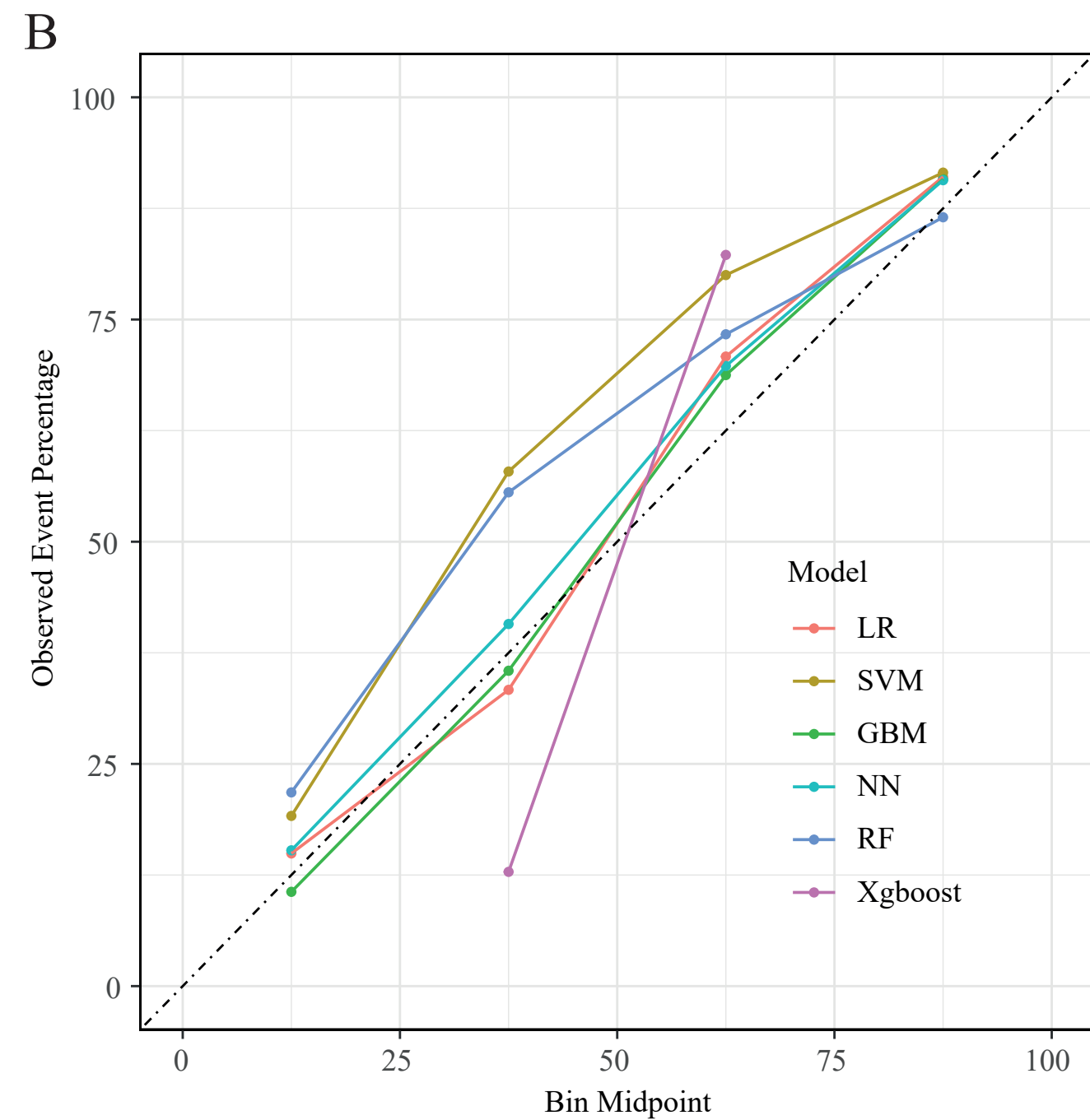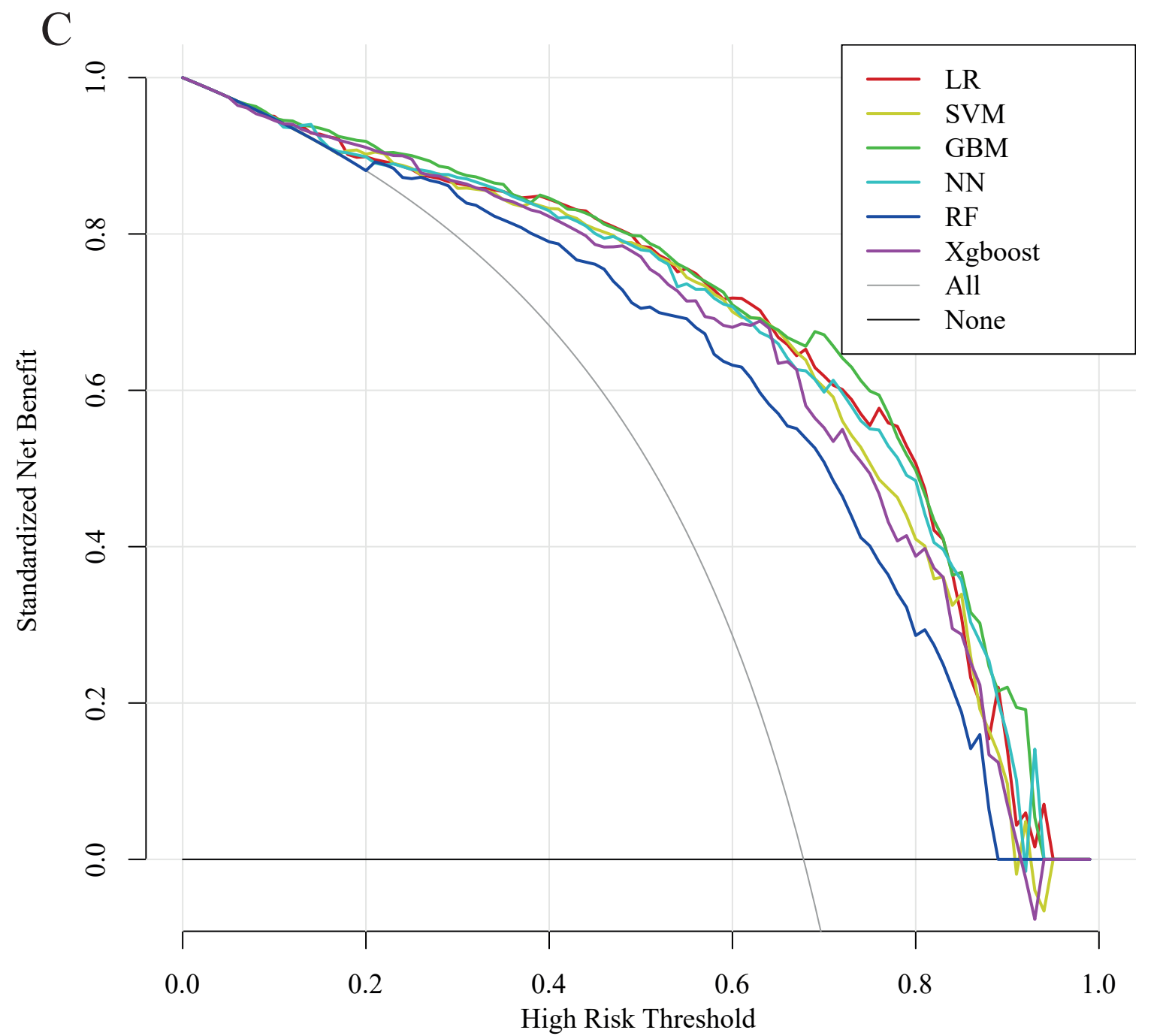

Supplement: Supplementary file 4 — Additional file 4. Fig S3: Calibration curves of six machine learning models. (A) Training set; (B) test set. Calibration curves for LR, RF, GBM, ANN, SVM, and XGBoost. The x-axis represents predicted probability, and the y-axis denotes observed incidence. The dashed line indicates perfect calibration, while colored lines reflect actual model performance. [file 12916_2025_4523_MOESM4_ESM.zip › Additional file 5/Fig. S4.pdf]

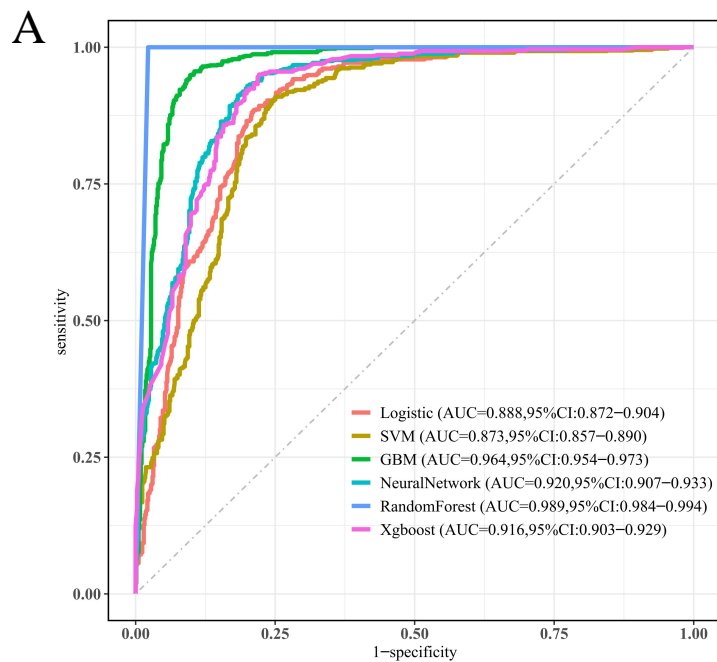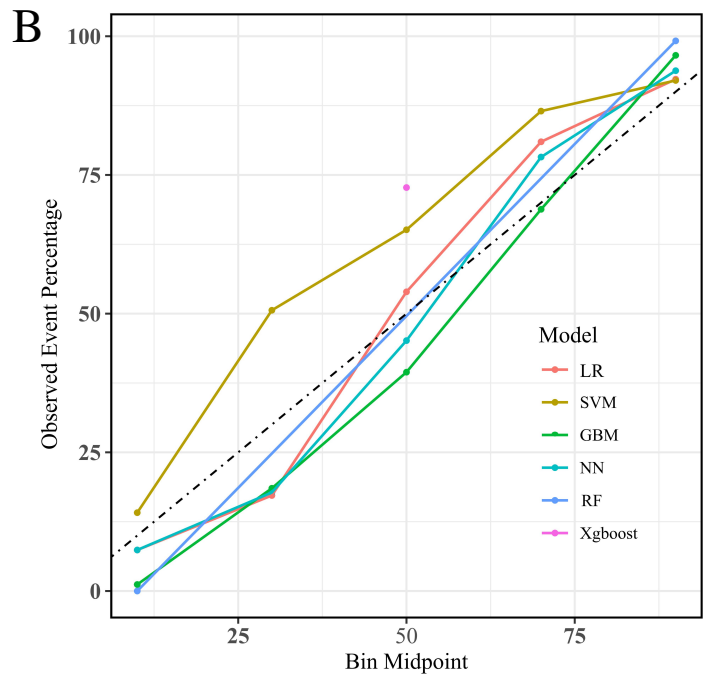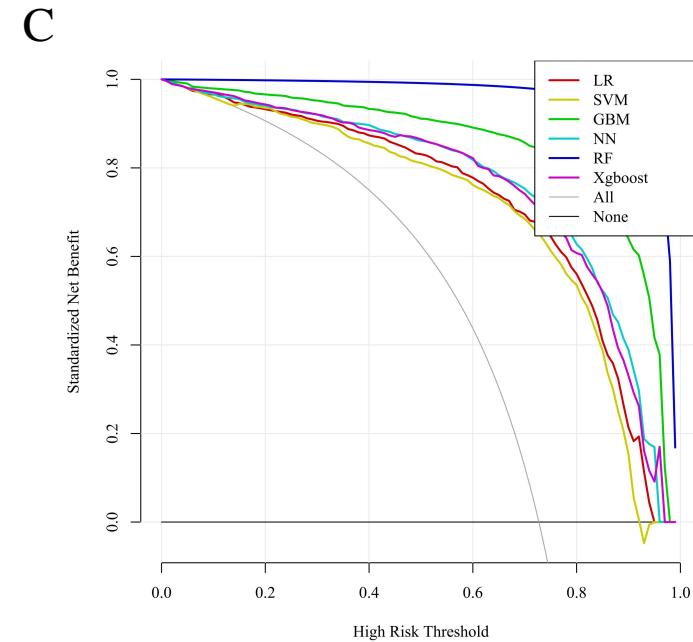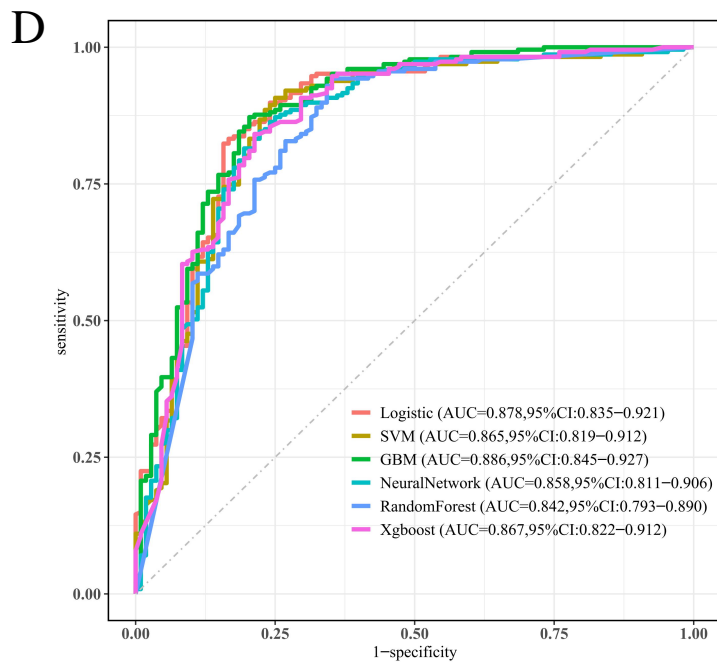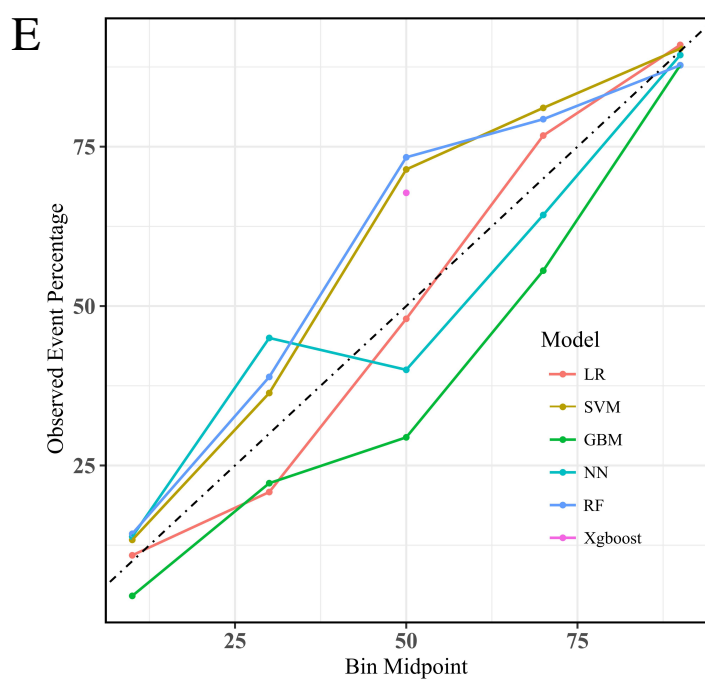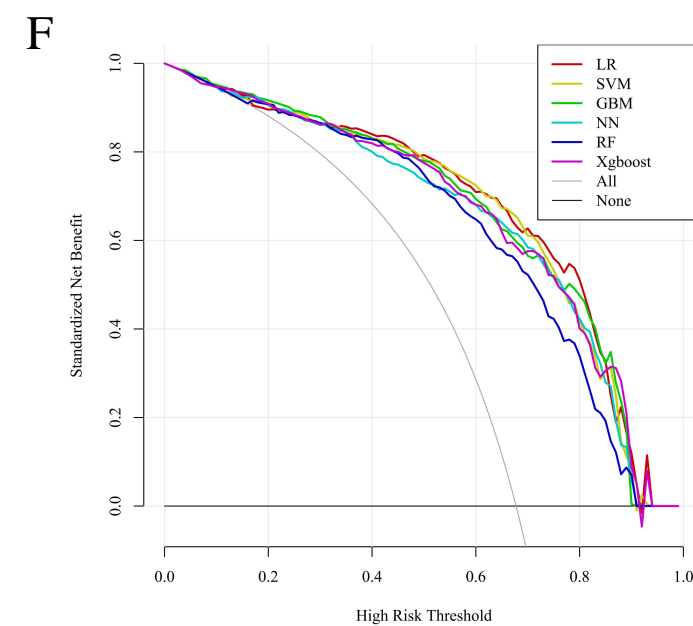

Supplement: Supplementary file 5 — Additional file 5. Fig S4: Model performance after SMOTE oversampling. (A-C) ROC, calibration, and decision curve analyses in the training set; (D-F) corresponding results in the test set. [file 12916_2025_4523_MOESM5_ESM.zip › Additional file 5/Fig S4.pdf]
